# Speciation and repeated origins of hypertrophied lips in parallel adaptive radiations of cyprinid fish from East Africa

**DOI:** 10.1002/ece3.10523

**Published:** 2023-09-12

**Authors:** Boris Levin, Aleksandra Komarova, Evgeniy Simonov, Alexei Tiunov, Marina Levina, Alexander Golubtsov, Fyodor Kondrashov, Axel Meyer

**Affiliations:** ^1^ Papanin Institute for Biology of Inland Waters Russian Academy of Sciences Yaroslavl Russia; ^2^ Zoological Institute of Russian Academy of Sciences Saint‐Petersburg Russia; ^3^ A.N. Severtsov Institute of Ecology and Evolution of the Russian Academy of Sciences Moscow Russia; ^4^ Eco‐Analytical Laboratory Cherepovets State University Cherepovets Russia; ^5^ Okinawa Institute of Science and Technology Okinawa Japan; ^6^ Department of Biology University of Konstanz Konstanz Germany

**Keywords:** Africa, ddRAD, fishes, parallel evolution, sympatry, trophic divergence

## Abstract

The evolution of convergent phenotypes is one of the most interesting phenomena of repeated adaptive radiations. Here, we examined the repeated patterns of thick‐lipped or “rubberlip” phenotype of cyprinid fish of the genus *Labeobarbus* discovered in riverine environments of the Ethiopian Highlands, East Africa. To test the adaptive value of thickened lips, identify the ecological niche of the thick‐lipped ecomorphs, and test whether these ecomorphs are the products of adaptive divergence, we studied six sympatric pairs of ecomorphs with hypertrophied lips and the normal lip structure from different riverine basins. Trophic morphology, diet, stable isotope (δ^15^N and δ^13^C) signatures, as well as mtDNA markers and genome‐wide SNP variation, were analyzed. Our results show that thick‐lipped ecomorphs partition trophic resources with generalized ecomorphs in only one‐half of the examined sympatric pairs despite the pronounced divergence in lip structure. In these thick‐lipped ecomorphs that were trophically diverged, the data on their diet along with the elevated ^15^N values suggest an invertivorous specialization different from the basal omnivorous–detritivouros feeding mode of the generalized ecomorphs. Genetic data confirmed an independent and parallel origin of all six lipped ecomorphs. Yet, only one of those six thick‐lipped ecomorphs had a notable genetic divergence with sympatric non‐lipped ecomorphs based on nuclear SNPs data (*F*_ST_ = 0.21). Sympatric pairs can be sorted by combinations of phenotypic, ecological, and genetic divergence from an ecologically non‐functional mouth polymorphism via ecologically functional polymorphism to a matured speciation stage via divergent evolution.

## Graphical abstract

Speciation continuum was revealed within repeated de‐novo origins of lipped mouth in African cyprinids. Diet, stable isotopes and genomics showed step‐by‐step moving from non‐functional polymorphism via ecologically functional polymorphism (trophic resource partitioning) to ecological speciation when genomic divergence occurred.

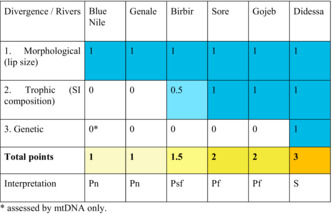

## 1 INTRODUCTION

Phenotypic variation provides the crucial basis for divergent selection to act upon and is the source for the further evolution of novel morphological and ecological diversity. Variation in trophic morphology is of particular interest to evolutionary biologists since the partitioning of trophic resources is considered one of the main prerequisites for ecological speciation and adaptive radiation (Meyer, 1987; Rüber et al., 1999; Rundle et al., 2000; Sibbing et al., 1998). Linking phenotypic traits to an adaptive ecological function requires information at different levels of biological organization. Moreover, it remains uncertain what is primary during adaptive radiation – a behavioral or ecological adaptation that precedes morphological specialization or morphological novelty promoting the filling of the new ecological niches (reviewed in Schluter, 2000). Adaptive radiations based on trophic resource partitioning have been studied in fishes, such as cichlids, coregonids, Arctic charr, three‐spined sticklebacks, and many other lineages of fish (Barluenga et al., 2006; Burress, 2016; Martin & Wainwright, 2011; Schluter, 2000; Seehausen & Wagner, 2014; Sibbing & Nagelkerke, 2000; Skulason & Smith, 1995). Cyprinid fishes, family Cyprinidae sensu *lato*, is one of the most diversified families of Actinopterygii (>3000 species – Fricke et al., 2022) that contains many adaptive radiations (e.g., Komarova et al., 2021; Kornfield & Carpenter, 1984; Levin et al., 2020; Levin, Simonov, et al., 2021; Mina et al., 1996; Nagelkerke et al., 1994; Savvaitova et al., 1987), most of which, however, have not yet been studied using genome‐wide approaches.

**FIGURE 1:**
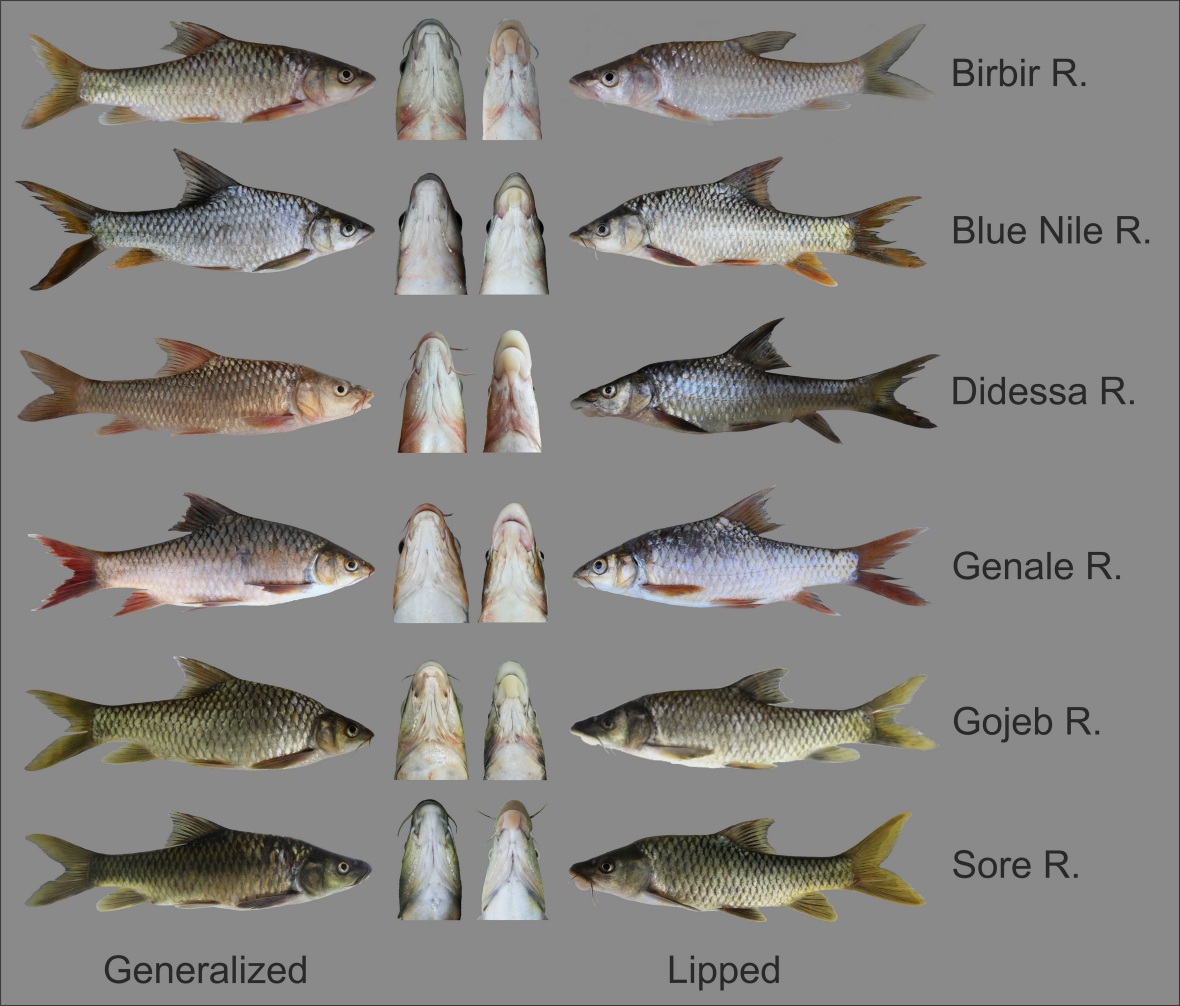
Phenotypes of generalized and thick‐lipped ecomorphs of the *Labeobarbus* spp. from rivers of the Ethiopian Highlands. Mature fish in “alive” coloration and their heads (bottom view) are shown.

**FIGURE 2:**
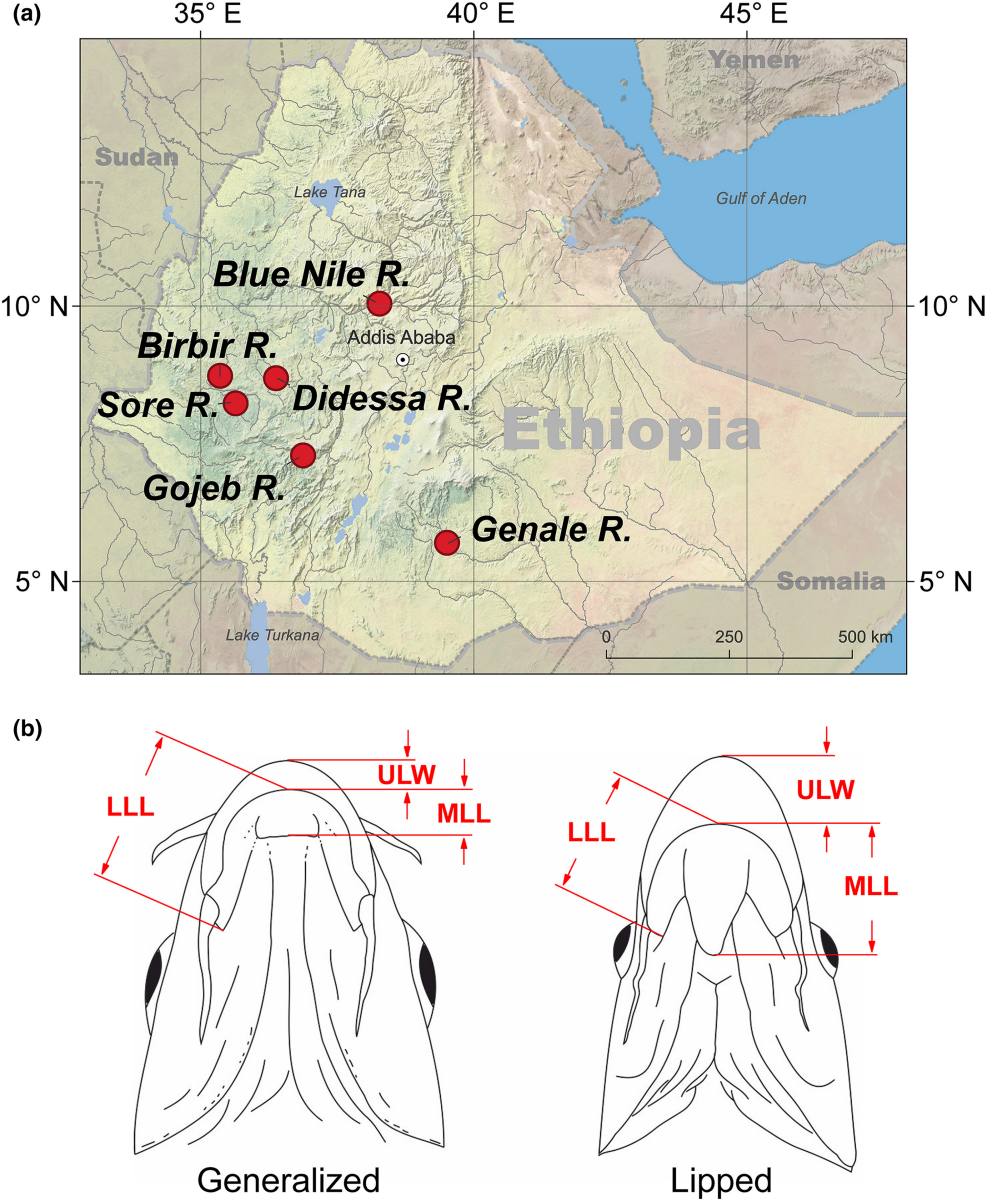
(a) Map with sampling sites of *Labeobarbus* sympatric pairs (generalized and thick‐lipped ecomorphs) from rivers of the Ethiopian Highlands; the map was created using QGIS v.3.16.4. (b) Scheme of lips measurements: LLL, lower lip length; MLL, middle lobe length; ULW, upper lip width.

Large African barbs of the genus *Labeobarbus* Rüppell, 1835, are a remarkably diverse lineage of polyploid cyprinid fishes (2n = 150 – Golubtsov & Krysanov, 1993; Oellermann & Skelton, 1990) with >130 species (Fricke et al., 2022) that display great diversity as well as the distinct polymorphisms in mouth phenotypes (Banister, 1973). This diversity in trophic morphology is part of the likely explanation for why in the genus *Labeobarbus* numerous adaptive radiations based on tropic resource partitioning evolved. Such radiations are found in both lacustrine (e.g., the Lake Tana radiation is composed of 15 ecomorphs/species) as well as riverine environments (Levin et al., 2019, 2020; Mina et al., 1996; Mironovsky et al., 2019; Nagelkerke et al., 1994). Four major trophic phenotypes among *Labeobarbus* have been described: (i) generalized; (ii) algae scraping (with subtypes); (iii) with hypertrophied lips (thick‐lipped); and (iv) large‐mouthed, or piscivorous (with subtypes; Banister, 1973; Levin et al., 2020; Mina et al., 1998; Nagelkerke et al., 1994; Vreven et al., 2016). Remarkably, very similar mouth phenotypes repeatedly evolved throughout the range of *Labeobarbus* in Sub‐Saharan Africa (Levin et al., 2013, 2020; Tsigenopoulos et al., 2010; Vreven et al., 2016). One of the trophic types – the thick‐lipped phenotype – is of particular interest to this study and is characterized by hypertrophied lips with a well‐developed fleshy lobe on the lower jaw, which looks like a fleshy appendage on the chin (Figures 1 and 2b). The feeding preferences of fishes with such hypertrophied lips have been comprehensively studied in cichlids of both African and American clades (e.g., Baumgarten et al., 2015; Colombo et al., 2013; Machado‐Schiaffino et al., 2017; Manousaki et al., 2013; Ribbink et al., 1983; Sowersby et al., 2021; Stiassny & Meyer, 1999; Torres‐Dowdall & Meyer, 2021).

The cichlid species or ecomorphs with hypertrophied lips evolved a new foraging strategy in rocky crevices thereby occupying a new ecological niche. In particular, hypertrophied lips increase suction power by sealing cracks and grooves and also might protect the head from injury from prey on hidden benthic organisms (Baumgarten et al., 2015; Oliver & Arnegard, 2010). Thickened lips may also allow these cichlid species to achieve higher numbers of taste receptors (Manousaki et al., 2013; Oliver & Arnegard, 2010; Schemmel, 1967). Apart from hypertrophied lips (also known as “rubberlip”), they display also other adaptive features (like narrow and pointed head shape, etc. – see also Franchini et al., 2014; Fruciano et al., 2016). The divergent evolution of the thick‐lipped phenotype in cichlids was accompanied by assortative mating assessed in the experiments (Kautt et al., 2020; Machado‐Schiaffino et al., 2017).

The thick‐lipped mouth phenotype is one of the most frequently occurring within the *Labeobarbus* lineage (Banister, 1973; Vreven et al., 2016). Some individuals have such greatly hypertrophied lips that pioneering investigator E. Rüppell assigned a new generic name to them based on this conspicuous trophic feature (Rüppell, 1835). Genetic data (mtDNA) support numerous parallel origins of the thick‐lipped phenotype among *Labeobarbus* (Decru et al., 2022; Levin et al., 2020; Tsigenopoulos et al., 2010). Nevertheless, the ecological role of the thick‐lipped ecomorphs in *Labeobarbus* is not yet known. The generalized trophic phenotype has mainly detritivorous–omnivorous diet (e.g., Levin, Komarova, et al., 2021; Levin, Simonov, et al., 2021; Matthes, 1963; Sibbing & Nagelkerke, 2000; Teshome et al., 2023). The feeding preferences or trophic position of the thick‐lipped ecomorph has been investigated so far for only two cases. One study (Sibbing & Nagelkerke, 2000) investigated the diet composition of the lacustrine‐lipped phenotype of *L*. cf. *intermedius* (known also as *L. nedgia*, Rüppell, 1835) in Lake Tana (East Africa, Ethiopia). Another study (Levin et al., 2019) examined nitrogen and carbon stable isotope signatures in the riverine lipped phenotype of *L. gananensis* from the Genale River (Ethiopia). Their results provided weak support for trophic resource partitioning between fish with hypertrophied and normally developed lips (generalized mouth). So, weak trophic resource partitioning between sympatrically co‐occurring ecomorphs with strikingly divergent trophic morphology turned out to be an intriguing finding. In general, whether such mouth structure transition is just non‐adaptive phenotypic variation within highly polymorphic *Labeobarbus* lineage or whether thick lips are an adaptive trait involved in trophic resource partitioning remains unclear so far. To address this question, similar cases in a broader taxonomic and geographical context have been studied by us. We could show that thick‐lipped ecomorphs evolved several times independently in several Ethiopian river basins. The hypertrophied ecomorphs were indistinguishable by mtDNA from sympatric ecomorphs with a generalized mouth phenotype (Levin et al., 2020).

Our goals were twofold. First, we aimed to test whether hypertrophied lips are adaptive within *Labeobarbus* spp. (*L. gananensis* (Vinciguerra, 1895) and *L. intermedius* (Rüppell 1835) complex) as judged by information on trophic resource partitioning, i.e., whether a thick‐lipped ecomorph occupies a separate trophic niche. To do this, we compared the trophic morphology, diet and stable isotope signatures in generalized and thick‐lipped forms. Second, we tested the hypotheses about the genetic divergence between and reproductive isolation within sympatric pairs of thick‐lipped and generalized small‐lipped ecomorphs based on genome‐wide genetic data obtained by double digest restriction‐site associated DNA (ddRAD).

## 2 MATERIALS AND METHODS

### 2.1 Study sites

Sampling was done under the framework of the Joint Ethiopian–Russian Biological Expedition (JERBE) in six rivers draining the Ethiopian Highlands and belonging to its four major river basins (Figure 2): (i) the White Nile basin – the Birbir R. – 8.7364° N 35.3518° E, and the Sore R. at two locations – at the City of Metu – 8.3178° N, 35.5951° E – and ~35 km downstream along the river course – 8.3987° N, 35.4378° E; (ii) the Blue Nile basin – Blue Nile at the City of Dejen – 10.0775° N 38.1934° E – and the Didessa R., a tributary of the Blue Nile – 8.6921° N 36.4144° E; (iii) the Omo‐Turkana enclosed basin – the Gojeb R., a tributary of the Omo R. – 7.2539° N 36.7943° E; (iv) the Juba‐Wabe‐Shebelle basin in the Indian Ocean catchment – the Genale R. – 5.7025° N 39.5446° E. Fish were caught by gill and cast nets in March 2014 (Birbir), February–March 2011 (Didessa), March–April 2009 (Genale), February 2011 (Gojeb), April 2014 (Sore), and January 2022 (Blue Nile). Fish were killed with an overdose of MS‐222 anesthetic and then photographed using a Canon EOS 50D camera (Canon Inc.). The standard length (SL, mm) was measured with a ruler. Fish were preserved first in 10% formalin and then transferred to 70% ethanol. All specimens are deposited at A.N. Severtsov Institute of Ecology and Evolution, the Russian Academy of Sciences, Moscow under provisional labels of JERBE. In total, up to 296 samples (morphology: *n* = 252; gut length: *n* = 296; diet: *n* = 81; stable isotopes: *n* = 237; mtDNA: *n* = 213; ddRAD: *n* = 63) were investigated by a set of various methods (Table S1).

### 2.2 Morphology

The head length (*C*), middle lobe length (*MLL*), lower lip length (*LLL*), and upper lip width (*ULW*) were measured in CorelDRAW 2017 (v. 19.1.0.419) using photographs. The scheme of measurements is given in Figure 2b. The indexes of *MLL*, *LLL*, and *ULW* to head length (*C*) were used for subsequent analyses. The standard length (SL) and gut length (GL) were measured on preserved specimens using a ruler (to the nearest 1 mm). A ratio *GL* to *SL* expressed as % was used for analyses. The SL distribution is given in File S2.

### 2.3 Diet

Gut content was dried on filter paper and weighed using a Pioneer PX84/E balance with 0.0001 g accuracy. The diet particles were identified using Olympus CX41 microscope (100–1000× magnification) and Motic DMW‐143‐N2GG stereomicroscope (100–400× magnification). The diet components were grouped into: (i) detritus, (ii) invertebrates, (iii) macrophytes and (iv) fish. The group “Invertebrates” included the larvae of amphibiotic insects, and their fragments. The group “Macrophytes” included any fragments of plants – such as leaves, stems or seeds. A composite measure of diet, an index of relative importance (*IR*; Natarajan & Jhingran, 1961; Popova & Reshetnikov, 2011), was used to assess the contribution of different components to the diet. The *IR* index was calculated as follows: $\mathrm{IR}=\;\frac{F_{i}\times P_{i}}{\sum \left(F_{i}\times P_{i}\right)}\times 100\%$, where *F*_*i*_ is the frequency of occurrence of each food group, and *P*_*i*_ is its part by weight; the value of *i* itself changes from 1 to *n* (*n* = the part of food organisms in the food bolus).

### 2.4 Stable isotope composition

For stable isotope (SI) analyses, white muscle tissue from the dorsal side of the body under the dorsal fin was sampled from freshly collected specimens. White muscle samples were dried at 60°C for subsequent SI analyses. The samples were weighed using a Mettler Toledo MX5 microbalance (Mettler Toledo) with 2 μg accuracy and were wrapped in tin capsules. The weight of the fish tissue samples varied from 250 to 500 μg. SI analysis was conducted at the Joint Usage Center of the A.N. Severtsov Institute of Ecology and Evolution RAS, Moscow. Briefly, a Thermo Delta V Plus continuous‐flow IRMS was coupled to an elemental analyzer (Flash 1112) equipped with a Thermo No‐Blank device. The isotopic composition of N and C was expressed in the δ notation relative to the international standards (atmospheric nitrogen and VPDB, respectively): δX (‰) = [(*R*_sample_/*R*_standard_) − 1] × 1000, where *R* is the molar ratio of the heavier isotope to the lighter. The samples were analyzed with a reference gas calibrated against the International Atomic Energy Agency (IAEA) reference materials USGS 40 and USGS 41 (glutamic acid). The measurement accuracy was ±0.2‰. Along with the isotopic analysis, the nitrogen and carbon content (as %) and C/N ratios were determined. In total, 237 white‐muscle samples were analyzed.

### 2.5 Statistical analyses of morphological and ecological data

Several R packages and functions were used for the statistical analyses and plot construction. Basal descriptive statistics were obtained using the *summarytools* library (Comtois, 2018). The Mann–Whitney *U* test was applied for pairwise comparison of the lipped and generalized ecomorphs in lip characters *MLL*, *LLL*, *ULW*, gut length *GL*, and SI composition within each locality using the function *wilcox*.*test* (in package *stats*; RStudio Team, 2021). The Pearson correlation and the violin boxplots were obtained using the *ggplot2* library (Wickham, 2016). Principal component analysis (PCA) was done using the *prcomp* function. For visualization of PCA results, the packages *factoextra* library (Kassambara & Mundt, 2020), *ggfortify* library (Tang et al., 2016), and *ggplot2* library (Wickham, 2016) were used. The package SIBER v.2.1.6 (Jackson et al., 2011) was used to assess the differences in the isotopic trophic niche features. The total convex hull areas (TA), core trophic niche breadths, and sample size‐corrected standard ellipse area (SEAc) were estimated. The trophic overlap for 95% TA was estimated using nicheROVER (Lysy et al., 2021), a method that is insensitive to the sample size and incorporates statistical uncertainty using the Bayesian approach (Swanson et al., 2015).

### 2.6 DNA sampling, extraction, amplification, sequencing, and analysis – mtDNA Data

DNA samples (*n* = 213) were collected from both generalized and lipped ecomorphs of *Labeobarbus* from the same six localities in Ethiopian Highlands (Figure 2b; Table S3 for details). Total genomic DNA was extracted from ethanol‐preserved fin tissues using the QIAamp DNA Micro kit (Qiagen). Sequences of the mitochondrial gene cytochrome *b* (*cytb*), 1038 bp in length, were amplified (polymerase chain reaction (PCR) conditions were taken from Palumbi, 1996; Perdices & Doadrio, 2001). PCR products were visualized on 1.5% agarose gels, purified with ExoSAP‐IT and sequenced using an ABI 3500 sequencer. Some sequences were obtained previously (Levin et al., 2020; GenBank accession Nos. are given in Table S3) while new sequences obtained in this study were deposited in GenBank under accession Nos. OQ604627‐OQ604649 (see Table S3 for details). All sequences were aligned and edited using the muscle algorithm (Edgar, 2004) as implemented in mega 6.0 (Tamura et al., 2013). The data final set is comprised of 213 *cytb* sequences. A haplotype network was constructed using the median joining algorithm (Bandelt et al., 1999) in popart 1.7 (Leigh & Bryant, 2015) with the default value of epsilon (0).

### 2.7 ddRAD‐seq library preparation

High‐molecular‐weight DNA was isolated from fin tissue preserved in ethanol using a QIAamp DNA Mini Kit (Qiagen) or obtained with a salt‐based DNA extraction method (Aljanabi & Martinez, 1997) followed by purification using a CleanUp Standard kit (Evrogen). The quantity of dsDNA was measured using a dsDNA HS Assay Kit for fluorometer Qubit 3 (Life Technologies). A ddRAD‐library was constructed following the quaddRAD protocol (Franchini et al., 2017) using restriction enzymes *Pst*I and *Msp*I. In total, 63 DNA samples of *Labeobarbus* ecomorphs from five riverine basins (see Table S1) were sequenced by two runs of Illumina HiSeq2500 and Illumina X Ten (2 × 150 bp paired‐end reads). The raw sequencing data from 63 samples were demultiplexed by the sequencing provider using outer Illumina TruSeq dual indexes and deposited at NCBI (BioProject ID PRJNA1000117).

### 2.8 Processing of RAD‐seq data

Read quality was assessed with fastqc 0.11.7 (Andrews & Krueger, 2010) and multiqc 1.13 (Ewels et al., 2016). Further demultiplexing of individually barcoded samples, construction and cataloging of RAD‐loci and single nucleotide polymorphism (SNP) calling were done with stacks 2.62 (Rochette et al., 2019). Identification and removal of PCR duplicates were done using the *clone_filter* module of stacks. The stacks module *process_radtags* was used to demultiplex reads by the dual index inner barcodes and obtain separate fastq files for each individual. Demultiplexed reads were trimmed at their 5′‐ and 3′‐ ends by 5 bp to a uniform length of 140 bp using fastp 0.20.1 (Chen et al., 2018) to reduce the influence of sequencing error (due to decreased base quality at both ends). Samples that failed to produce more than 100,000 reads were excluded from further processing. The *integrated* approach of stacks was used to assemble loci de novo and perform genotype calling after mapping assembled loci to a heterospecific high‐quality reference genome of *Barbus barbus* (GenBank assembly accession: GCA_936440315.1) and filtering. We selected optimal parameters for de novo loci assembly using the approach suggested by Paris et al. (2017). Following the aforementioned procedure, we found that a minimum stack depth (−*m*) of 6, distance allowed between stacks (−*M*) of 1 and maximum distance required to merge catalog loci (−*n*) of 1 provided the best balance between data quality and quantity for our data set. We also set the ‐‐max_locus_stacks parameter to 7 to improve binning and avoid paralogs (Stobie et al., 2018). We align the loci catalog generated in the de novo assembly to the reference genome using bwa mem v.0.7.17 (Li & Durbin, 2009) with default settings. To avoid spurious alignments of de novo loci, “stacks‐integrate‐alignments” was run with minimum alignment coverage and percent identity both set to 0.8.

### 2.9 Population genomic and phylogenomic analyses

To test whether sympatric ecomoprhs of *Labeobarbus* from different basins are independent evolutionary units we used maximum likelihood phylogenetic analysis based on SNPs in iq‐tree 1.6.12 (Minh et al., 2020). Multiple sequence alignment (MSA) of SNPs were created using the “‐‐phylip‐var” option of the *populations* module of stacks with retention of loci genotyped in at least 70% of all samples and SNPs with a minor allele count above 3. Heterozygous sites within each individual were encoded using IUPAC notation. Invariant sites (arising due to missing data) were excluded from the MSA by IQ‐TREE, resulting in 15,820 nucleotide sites. To take into account the absence of constant sites an ascertainment bias correction (+ASC) model (Lewis, 2001) was applied to all substitution models in a best‐fit model selection process with ModelFinder (REF). Branch support values were obtained using an ultrafast bootstrap procedure (Hoang et al., 2018) with 1000 replicates. The phylogenetic tree was visualized using figtree 1.4.4 (Rambaut, 2014).

STRUCTURE 2.3.4 (Pritchard et al., 2000) was used to examine the population structure of the whole dataset and within each pair of ecomorphs (i.e. basin). First, all individual genotypes were filtered and tested for deviations from Hardy–Weinberg Equilibrium (HWE) using the *populations* module of stacks with the following settings: (i) loci genotyped in at least 80% of all samples were kept; (ii) SNPs with a minor allele count (‐‐min‐mac) less than 3 were pruned. Next, we whitelisted loci that are in HWE for all populations and created a structure‐formatted file with *populations* while retaining a single random SNP per locus to avoid the inclusion of closely linked SNPs. This dataset consisted of 2411 SNPs. We performed 16 independent runs (100,000 chains as burn‐in plus 100,000 MCMC chains) of STRUCTURE for each *K* between 1 and 8 using the admixture model with correlated allele frequencies. Structure_threader (Pina‐Martins et al., 2017) was used to parallelize the runs. Results of the Structure runs were summarized using CLUMPAK webserver (Kopelman et al., 2015) with default settings. The optimal *K*‐value was determined by the approaches of Pritchard et al. (2000) and Evanno et al. (2005). The same protocol was followed for consecutive hierarchical STRUCTURE runs for the identified clusters until no subdivision was revealed (i.e., *K* = 1). For these runs, we created new SNP sets using the same procedure as described above but limiting genotypes to the individuals from identified genetic clusters. In addition, Principal Component Analysis (PCA) was performed using the glPca function of the ADEGENET 2.1.1 R‐package (Jombart & Ahmed, 2011). Pairwise Reich–Patterson *F*_ST_ values (Reich et al., 2009) with respective 95% confidence intervals for ecomorphs/genetic clusters were calculated using the R script from Junker et al. (2020). The PCA and Reich–Patterson *F*_ST_ calculations were done on the aforementioned 2411 SNPs dataset.

## 3 RESULTS

### 3.1 Morphology

#### 3.1.1 Lip size

**FIGURE 3:**
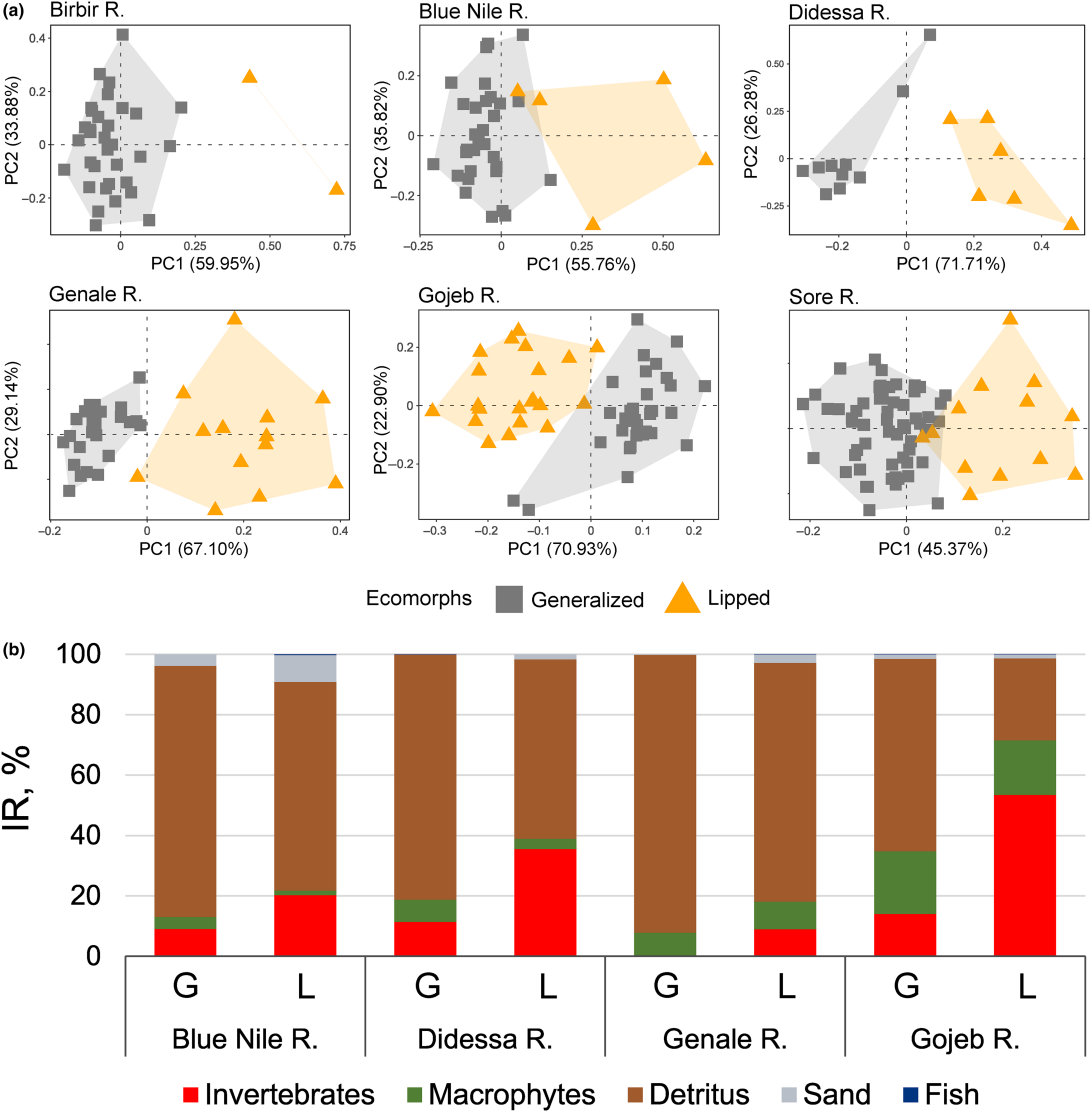
(a) PCAs of sympatric pairs of generalized versus thick‐lipped ecomorphs of *Labeobarbus* spp. that live sympatrically in each of six rivers: Birbir, Blue Nile, Didessa, Genale, Gojeb, and Sore. (b) Food spectra (IR: the index of relative importance) of the sympatric ecomorphs (G, generalized; L, thick‐lipped) of the *Labeobarbus* spp. from the Blue Nile, Didessa, Genale, and Gojeb.

The principal component analysis confirmed a large divergence between sympatric pairs of lipped and generalized ecomorphs in PC1 and PC2 space in all studied rivers (Figure 3a). In three of the six ecomorph comparisons (Birbir, Didessa, and Genale), the nonoverlap was detected and the distributions in PC‐space were only weakly overlapping for the other three comparisons of sympatric ecomorphs (Blue Nile, Gojeb, and Sore). PC1 explained from 45.4% (Sore R.) to 71.7% (Didessa R.) of the variance, while PC2 explained less than 36%. Eigenvectors of the characters for all PCs are given in Table S4. The PCA of both ecomorphs from all rivers is given as File S5. Middle lobe length (MLL) and upper lip width (ULW) had the highest contribution to divergence of sympatric ecomorphs.

The thick‐lipped ecomorph was characterized by greatly developed middle lobes on the lower lip (MLL) that were significantly longer compared to the generalized ecomorph in all studied rivers (*p* < .01; Figure S6). Individual MLL values varied from 10% to 33% (as % head length) in the thick‐lipped ecomorph (averaging – 21.32 ± 0.70 SE) while in the generalized ecomorph, they were much lower – from 4% to 16% (averaging – 8.53% ± 0.13 SE). The largest MLL values within thick‐lipped ecomorph were detected in the Birbir River (27%) while the smallest ones – in the Sore River (16%) and Blue Nile River (16%).

#### 3.1.2 Gut length

The guts of thick‐lipped ecomorphs were shorter than in generalized ecomorphs from all studied rivers, sometimes significantly (Figure S6). The gut length of thick‐lipped ecomorph varied from 140.0% to 507.8% SL, averaging – 305 ± 8.5 SE. The gut length of generalized ecomorph varied from 157.2% to 646.1%, averaging – 333 ± 5.6 SE. Significant differences between sympatric thick‐lipped and generalized ecomorphs were detected in the Blue Nile (*p* < .01), Genale (*p* < .05) and Gojeb (*p* < .01; Wilcoxon test).

### 3.2 Trophic divergence

#### 3.2.1 Diet

Food spectra of sympatric ecomorphs of *Labeobarbus* spp. were rather diverse and composed of (i) detritus, (ii) invertebrates (mainly insects: Ephemeroptera, Hemiptera, Trichoptera, Coleoptera, Hymenoptera, and Diptera), (iii) macrophytes (remnants of helophytic and semi‐aquatic plants, represented by seeds, leaves, stems, and flower parts, sometimes coupled with filamentous algae), and occasionally (iv) fish (juvenile parts or scales). Feeding of sympatric ecomorphs in all four rivers studied was divergent but at various degrees as estimated by the index of relative importance (Figure 3b).

Detritus was the main component among food items in both generalized (63.8%–92.1%) and thick‐lipped ecomorphs (27.1%–79.0%; Figure 3b). Generally, the diet of the thick‐lipped ecomorph was more insectivorous compared to the generalized ecomorph in all rivers studied (Figure 3b). Invertebrates in the diet of both ecomorphs were represented by larvae of Ephemeroptera and Trichoptera, larvae and imagoes of Diptera and Coleoptera as well as imagoes of Hemiptera, and Hymenoptera. The contributions of invertebrates for fish feeding might be underestimated due to their quick digestion. This is why we also provide data on N and C stable isotope composition (see below). A detailed description of the diet of each ecomorph in certain rivers is given in Supplementary material S7 in Appendix S1.

#### 3.2.2 Stable isotope composition

**FIGURE 4:**
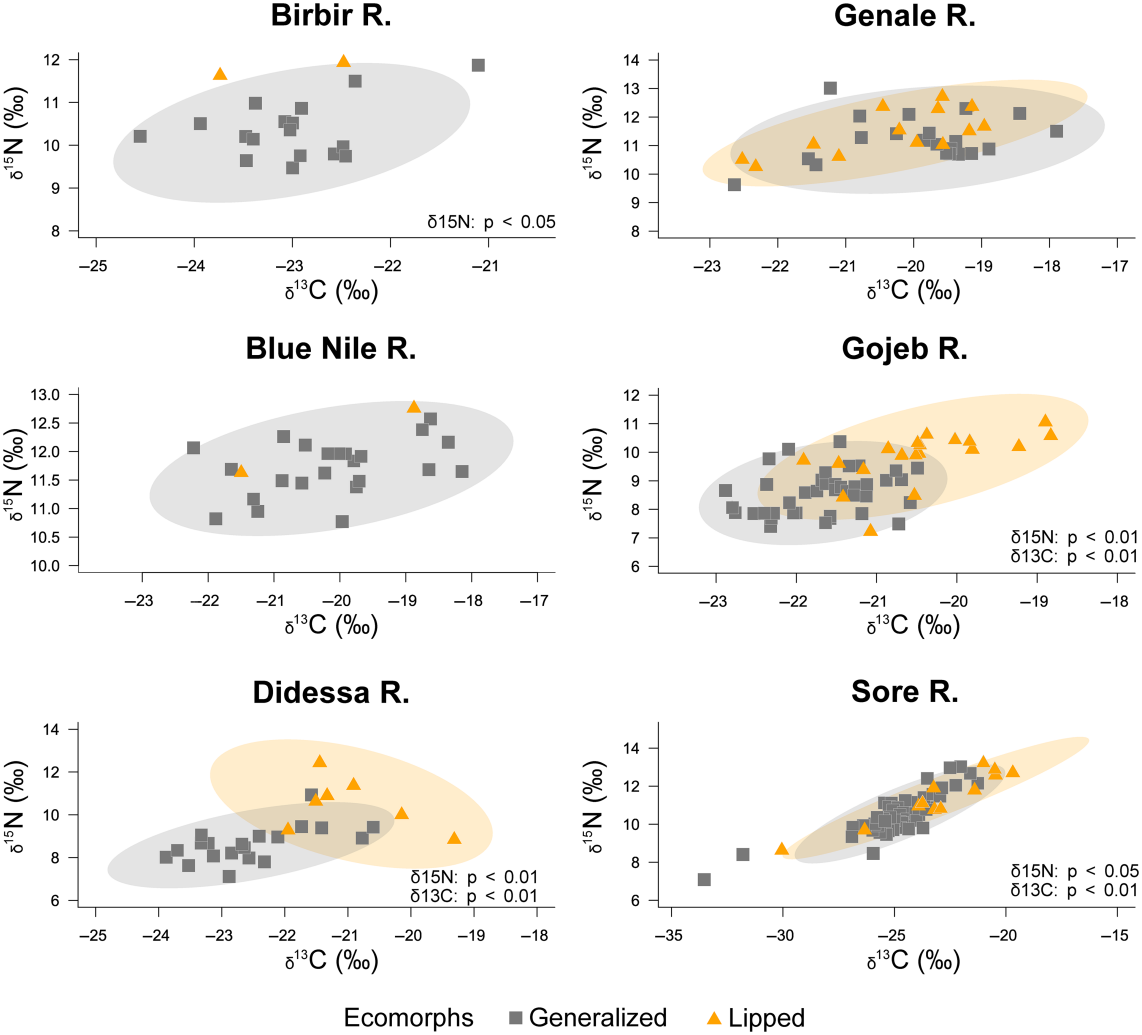
SI Bayesian ellipses showing trophic niche widths and overlaps in sympatric ecomorphs of the *Labeobarbus* spp. from the Birbir, Blue Nile, Didessa, Genale, Gojeb, and Sore Rivers. Ellipses with 95% confidence intervals are based on standard ellipses corrected for small sample sizes (SEAc; isotopic niche metrics; SIBER package). Each point corresponds to the isotopic value.

Basic statistics for both δ^15^N and δ^13^C values are given in Table S8. The thick‐lipped ecomorph had higher δ^15^N values than the generalized one in all comparisons with statistically significant differences in four of six paired comparisons: Birbir, *p* < .05; Didessa, *p* < .01; Gojeb, *p* < .01; and Sore, *p* < .05 (Figure 4 and Figure S9). When significant, the difference in mean δ^15^N values between sympatric generalized and thick‐lipped ecomorphs varied from 1.9‰ (Didessa) to 0.9‰ (Sore). Significant differences in δ^13^C values between sympatric pairs were found in the Didessa, Gojeb, and Sore Rivers. In all these cases, the lipped ecomorph had higher δ^13^C values (the difference varied from 1.6‰ to 1.2‰).

The total area (TA), standard ellipse area (SEA), and corrected standard ellipse area (SEAc) were analyzed for all sympatric ecomorphs except for thick‐lipped ecomorphs from the Blue Nile and Birbir Rivers because of the small sample size. The thick‐lipped ecomorph had the largest SEAc values in all rivers apart from the Genale River (Table S10). The niche overlap between generalized and thick‐lipped ecomorphs consisted of 25.7% in the Didessa River, 57.2% in the Gojeb River, and was considerably larger in the Sore and Genale Rivers (81.6% and 82.3%, respectively; Table S11).

### 3.3 Mitochondrial DNA divergence

**FIGURE 5:**
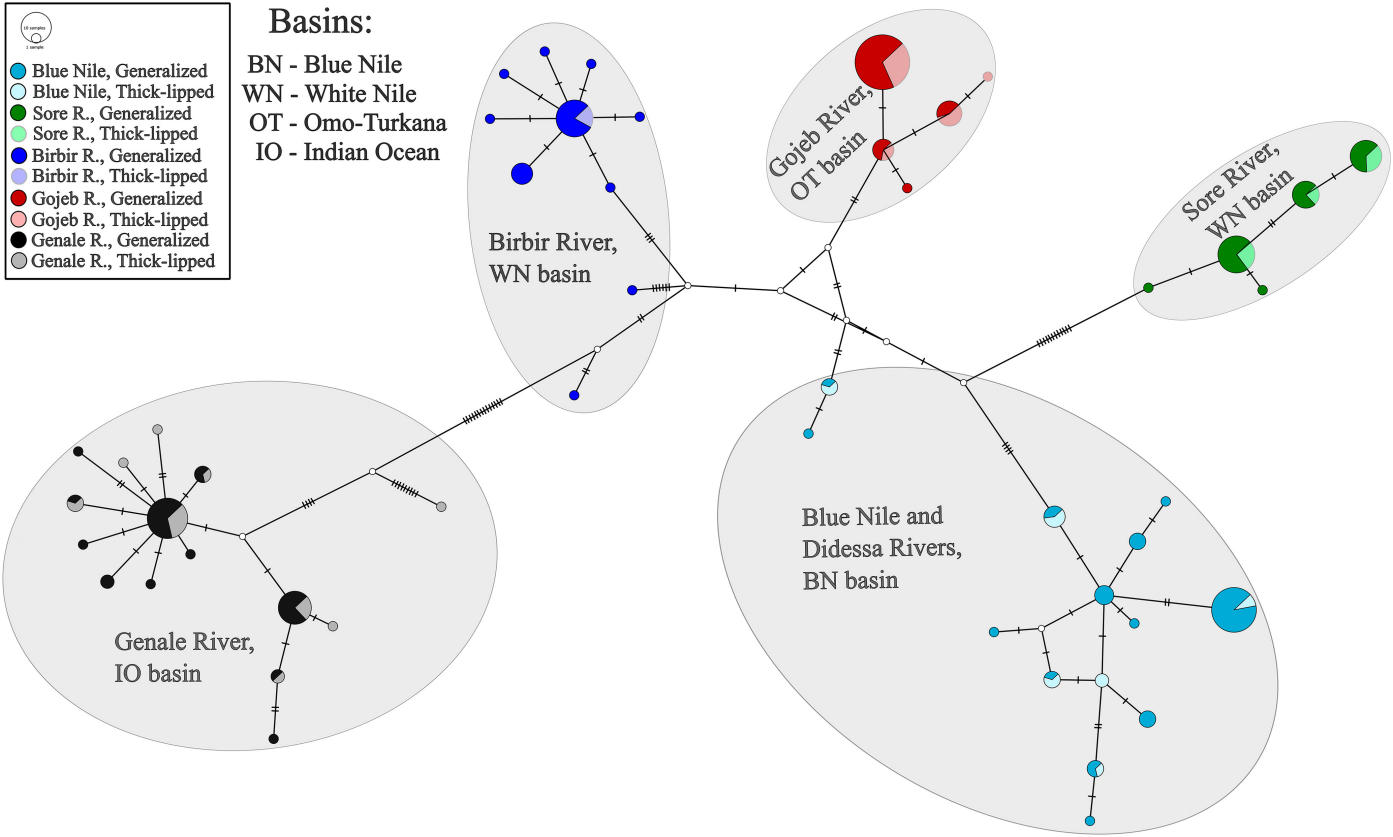
Median‐joining haplotype network of the generalized and thick‐lipped ecomorphs of the *Labeobarbus* spp. from four main drainages of the Ethiopian Highlands constructed on the basis of 213 cyt*b* sequences. Haplotypes of the generalized ecomorphs are colored more intensively in each case of sympatry.

Among the 213 individuals sampled, 49 different haplotypes were detected. The haplotype network is complex (Figure 5) and composed of five main haplogroups that correspond to (i) Blue Nile basin (including the Didessa R.), (ii) Genale R. (Indian Ocean basin), (iii) Gojeb R. (Omo‐Turkana basin), (iv) Birbir R. (White Nile basin), and (v) Sore R. (White Nile basin; Figure 5). A few samples from Didessa R. and Birbir R. were close to central putative haplotypes interconnecting the analyzed haplogroups. Genetic *p*‐distances between the geographic basins varied from 0.009 ± 0.003 to 0.031 ± 0.005 (Table S12). No haplotype sorting between sympatric generalized and thick‐lipped ecomorphs within each basin was revealed (Figure 5).

### 3.4 Phylogenetic relationships and genetic population structure inferred from the nuclear genome

Among 595,163 de novo catalog loci generated with STACKS de novo pipeline and mapped to the *B. barbus* genome 251,956 loci had multiple alignments, 322,820 had one alignment and 20,387 were unmapped. The “stacks‐integrate‐alignments” procedure filtered out 371,899 loci due to mapping quality, 97,561 due to alignment coverage, and 12,020 due to percent identity, resulting in 93,296 loci retained for downstream analyses (detailed statistics on each sample including raw reads is given in Supplementary material S113 in Appendix S1).

**FIGURE 6:**
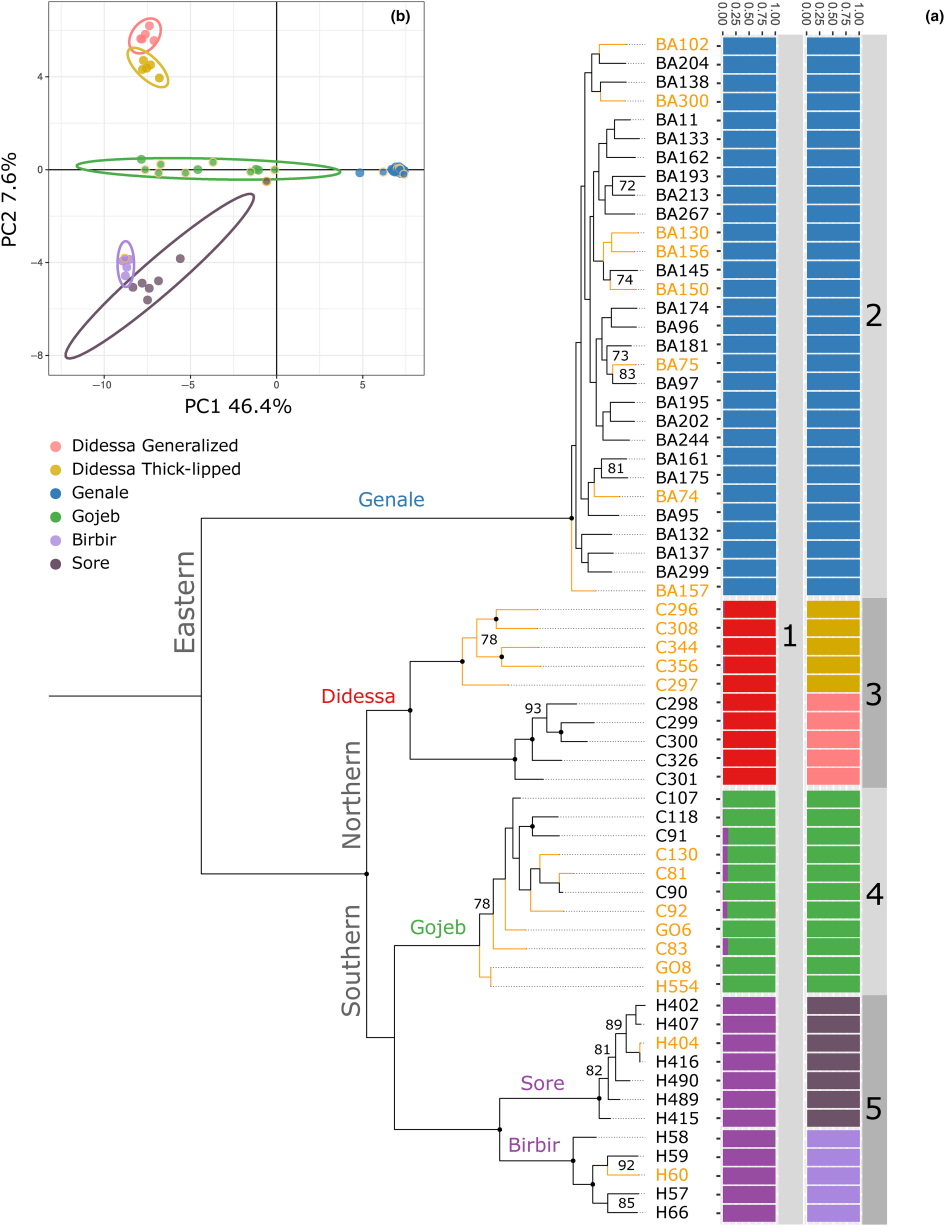
(a) Maximum likelihood SNP‐based phylogeny and genetic structure of generalized and thick‐lipped ecomorphs of *Labeobarbus* from the different basins of Ethiopia (Genale, Didessa, Gojeb, Sore, and Birbir Rivers). The tree inferred with IQ‐TREE2 using 15,820 variant SNPs with the TVM + F + ASC + R3 substitution model. Heterozygous sites within each individual were encoded using IUPAC notation. The thick‐lipped individuals in the tree are depicted in yellow color. Individuals are labeled by their voucher numbers. Ultrafast bootstrap values above 70% are shown as numbers near the corresponding nodes, while the black points in the nodes designate 95%–100% support. The genetic cluster proportions inferred by hierarchical STRUCTURE analysis are shown to the right of sample numbers: 1 – analysis of all samples with 2411 SNPs revealed best *K* = 4; 2 – analysis of the Genale genetic cluster with 7905 SNPs revealed no further subdivision; 3 – analysis of the Didessa genetic cluster with 6932 SNPs revealed subdivision on two clusters (best *K* = 2) corresponding to mouth phenotypes; 4 – analysis of the Gojeb genetic cluster with 4471 SNPs revealed no further subdivision; 5 – analysis of the Sore/Birbir (White Nile basin) genetic cluster with 2020 SNPs revealed subdivision on two clusters (best *K* = 2) corresponding to the river basins. (b) Principal Component Analysis (PCA) plot with points representing individuals and 95% confidence intervals colored by genetic clusters inferred with STRUCTURE. The thick‐lipped points are encircled in yellow accordingly to their coloration on the tree.

The phylogeny of Ethiopian *Labeobarbus* (Figure 6a) based on 15,820 SNPs is generally congruent with that based on mtDNA data (Levin et al., 2020 and Figure 6a). Sympatric generalized and thick‐lipped ecomorphs clustered together and form monophyletic lineages with high support for all studied basins/subbasins (90–100 bootstrap values) except for the Gojeb (62). The *Labeobarbus* lineages are subdivided into two clades representing (i) Eastern (*L. gananensis* from Genale River, Indian Ocean basin) and (ii) Western (*L*. cf. *intermedius* sensu lato from the Didessa, Gojeb, Sore, and Birbir Rivers) parts of the Ethiopian Highlands (Figure 6a). The сlade of the Western Plateau is further subdivided into the Northern subclade (Didessa River in the Blue Nile basin) and the Southern subclade that combines the Gojeb (Omo‐Turkana basin), Sore, and Birbir (White Nile basin) populations. The Southern subclade is further subdivided into lineages according to geographical basin belonging to the Gojeb (Omo‐Turkana) lineage and White Nile lineage comprising the Sore and Birbir populations. Remarkably, the Sore and Birbir populations also form the sister monophyletic sublineages within the White Nile lineage (Figure 6a).

An analysis of the population genetic structure revealed an optimum of four (*K*) clusters that correspond to the (i) Genale, (ii) Didessa, (iii) Gojeb, and (iv) White Nile populations from the Sore and Birbir Rivers (Figure 6a). Five samples from the Gojeb River have a little admixture (~10%) from the White Nile cluster. When each basin with sympatric ecomorphs was analyzed independently, only one river with subdivision by ecomorphs was revealed – the Didessa in the Blue Nile basin (Figure 6a). Individuals from the White Nile basin are further subdivided by geographical populations from the Sore and Birbir Rivers (Figure 6a). PCA of the 2411 SNPs confirmed six well‐defined clusters that correspond to the phylogenetic and population genomics results including subdivision of generalized and thick‐lipped ecomorphs in the Didessa (Figure 6b).

All Reich *F*_ST_ pairwise comparisons between inferred genetic clusters were statistically significant with values ranging from 0.21 (0.18–0.25 95%CI) between Didessa generalized ecomorph and Didessa thick‐lipped ecomorph to 0.66 (0.63–0.68) between Sore and Genale (Data S14). *F*_ST_ pairwise comparisons of the pairs of sympatric ecomorphs within each basin (Data S15) were significant only for Didessa, where ecomorphs constitute well‐segregated genetic pools.

## 4 DISCUSSION

The obtained results show that six cases of independently evolved thick‐lipped *Labeobarbus* ecomorphs from the Ethiopian Highlands are within a continuum from phenotypic polymorphism via trophic resource partitioning to ecological speciation.

### 4.1 Thick‐lipped mouth and trophic divergence

Although sympatric generalized and thick‐lipped ecomorphs were divergent in lip size in all six pairs, not all have partitioned trophic resources as might be expected from their phenotypic divergence. Generally, the diet of lipped ecomorph was enriched with benthic invertebrates compared to that of the generalized ecomorph with normally developed lips. However, the difference in the amount of consumed benthic invertebrates between lipped and non‐lipped ecomorphs varied greatly in different rivers. As one might expect, the sympatric pairs with larger differences in diet also showed significant divergence in SI signatures (e.g., in the Didessa and Gojeb) that confirmed the usage of SI composition as a diet proxy. Lipped ecomorphs had higher δ^15^N values compared to the generalized ecomorphs in all six comparisons (extra 0.2‰–1.9‰). Enrichment of the lipped ecomorph in δ^15^N (4 pairs: extra 0.9‰–1.9‰) was usually accompanied by enrichment in δ^13^C (3 of 4 pairs: extra 1.3‰–1.7‰). Nevertheless, SI Bayesian ellipses showed great trophic niche widths and overlaps in half of the comparisons of pairs of sympatric ecomorphs (Figure 4). When thick‐lipped ecomorph partitioned trophic resource with co‐occurring generalized ecomorph, it was more specialized to feed on aquatic invertebrates. But the variation in diet divergence between sympatric pairs of thick‐lipped and generalized *Labeobarbus* ecomorphs was rather large and some pairs did not show diet difference. In this regard, the parallel cases of the *Labeobarbus* are similar to other cases of sympatrically co‐occurring thick‐lipped and thin‐lipped cichlid fish such as ecomorphs/species of the genus *Amphilophus* Agassiz, 1859 from various Nicaraguan lakes (Elmer et al., 2010; Kautt et al., 2012; Manousaki et al., 2013).

Thus, hypertrophied lips in the *Labeobarbus* as a phenotypic trait are not sufficient to predict the diet of fish. The same was found not only for riverine populations but also for lacustrine. For instance, the thick‐lipped ecomorph in Lake Tana had almost the same food spectrum as sympatric generalized ecomorph (Sibbing & Nagelkerke, 2000). This is within Liem's paradox, i.e., that even species with specialized trophic morphologies have dietary flexibility (Liem, 1980). Many examples corroborate Liem's paradox suggesting it is a common phenomenon among fishes (e.g., Binning et al., 2009; Golcher‐Benavides & Wagner, 2019; Robinson & Wilson, 1998; Sturmbauer et al., 1992; Wagner et al., 2009). This phenomenon reduces the prediction of diet by phenotype and provides evidence for the greater trophic plasticity of specialists (see also recent examples from scraping feeders in Komarova et al., 2021, 2022). Previous experimental studies on various diets exposed to Neotropical and East African cichlid species showed outstanding phenotypic plasticity provoked by diet (Meyer, 1987; Muschick et al., 2014; Schneider et al., 2014). It suggests that not only certain phenotypes considered as trophically specialized maybe plastic in relation to diet but that phenotypic plasticity may also rise from a different diet. This coincides with a flexible stem hypothesis on the origin of adaptive radiations from ancestral flexible stems (Gibert, 2017; Schneider et al., 2014; Schneider & Meyer, 2017; West‐Eberhard, 2003; Wund et al., 2008).

What conditions can enable a thick‐lipped mouth to function? Both abiotic (e.g., type of bottom substrate etc.) or biotic (e.g., food resources availability) factors might promote the adaptive value of the thick‐lipped phenotype in some studied localities. Unfortunately, information on the phenotype‐environment correlation is hard to collect for thick‐lipped *Labeobarbus*. Moreover, existing data on the association of thick‐lipped phenotype with bottom substrate are unclear. Some studies reported stony habitats as preferred (Groenewald, 1957; Matthes, 1963), while another study (Kisekelwa et al., 2021) reported that species *L. longifillis* with thick‐lipped phenotype (Congo basin) appears to be linked to muddy substrates without pebbles, cobbles and boulders but in warmer localities with a relatively low electrical conductivity compared to sympatrically co‐occurring species with a “generalized” mouth. The presence of the thick‐lipped ecomorph on the muddy substrate is reported by F.N. Shkil (personal communication) for the Lake Tana basin. Due to an unstable hydrological regime in the mountain rivers of the Ethiopian Highlands, the environment varies largely from season to season. Our assessment of diet and trophic position using SI analyses was done based on the material collected during the dry season while the conditions may change during the wet season. One may suggest that thick‐lipped trophic morphologies may serve as a trade‐off between consumption of commonly available food (detritus) and benthic invertebrates in unstable riverine environments, e.g., when seasonally common prey is rare or even absent in some seasons. Additional ecological (natural and experimental) studies are needed for the clarification of the mechanisms of ecological functionalization of thick‐lipped phenotype.

### 4.2 Evolution of adaptive divergence: from repeated polymorphisms to ecological speciation

This study provides the first genome‐wide results on the phylogeny and population genetic structure of *Labeobarbus* from East Africa (genome‐wide data were previously obtained only for South African *Labeobarbus* by Stobie et al., 2018). Our phylogenetic results are in agreement with previously obtained mtDNA phylogenies (Beshera & Harris, 2014; Levin et al., 2020) and confirmed that thick‐lipped ecomorphs have evolved independently several times. The repeated origins of thick‐lipped ecomorphs in the river systems of the Ethiopian Highlands suggest that this polymorphism in trophic morphology might be an old developmental and genetic system that might be facilitated by genetic assimilation (Gunter et al., 2017; Schneider et al., 2014). Although results on genetic population structure confirmed the absence of genetic divergence between sympatric ecomorphs with and without hypertrophied lips in the most of populations, one sympatric pair was genetically divergent (in the Didessa River). To sort out the cases of the sympatric pairs in terms of polymorphism or divergent evolution, we analyzed data on trophic morphology, trophic niche segregation along with genetic data (Table 1).

**TABLE 1:** Heat‐map of ranking the sympatric pairs of generalized and thick‐lipped ecomorphs in different rivers based on divergence in trophic morphology, SI composition, and genetics: 0 – non‐significant difference; 1 – significant difference (0.5 is given for SI composition if only one of two analyzed elements is different).

Divergence/Rivers	Blue Nile	Genale	Birbir	Sore	Gojeb	Didessa
1. Morphological (lip size)	1	1	1	1	1	1
2. Trophic (SI composition)	0	0	0.5	1	1	1
3. Genetic	0^a^	0	0	0	0	1
**Total points**	**1**	**1**	**1.5**	**2**	**2**	**3**
Interpretation	Pn	Pn	Psf	Pf	Pf	S

*Note*: The populations were placed from left to right according to their total ranking from lower value to higher. Cells in rows of variables are colored according to points: 0 – no color, 0.5 – blue, 1 – intense blue; the “Total points” row is marked by various intense yellow color corresponded to a number of cumulative points (in bold) from 1 to 3 (1 ‐ half‐transparent yellow, 2 ‐ intense yellow, 3 ‐ orange; 1.5 points has an intermediate color between half‐transparent and intense yellow).

Abbreviations: P, polymorphism (n, ecologically non‐functional; sf, ecologically semi‐functional; f, ecologically functional); S, speciation event.

^a^ Assessed by mtDNA only.

The cases of divergence between sympatric ecomorphs in various rivers are ranked from 1 to 3 points. The results can be treated as a row of situations from ecologically non‐functional polymorphism (1 point) via ecologically functional polymorphism (2 points) to divergent evolution or speciation event (3 points in the presence of genetic divergence). The case of the Sore and Gojeb (2 points) showing the morphological and trophic divergence but no genome‐wide divergence can be considered an initial stage of speciation with only a few loci involved. Studies on genomic differentiation of thick‐lipped phenotype in *Amphilophus* cichlids from Nicaraguan lakes showed that only a small set of loci are responsible for this phenotype (Kautt et al., 2012, 2020; Machado‐Schiaffino et al., 2017; Sowersby et al., 2021). As for a sequence of divergence events, the evolution of thick‐lipped species of *Labeobarbus* might be realized into three steps: (i) rise of phenotypic divergence (most likely predetermined by ancient genetic polymorphism), (ii) rise of ecological (trophic) divergence that is based on the phenotypic divergence, (iii) genetic divergence caused by divergent selection upon ecological differentiation. At the last stage when all three types of divergence (phenotypic, ecological, and genetic) begin to be detectable, a species status is achieved. Accordingly, the sympatric pair from the Didessa River can be considered two different species. Notable divergence in population genomic structure based on ddRAD SNPs in the lack of sorting in mtDNA suggests a rather recent speciation in the case of the Didessa River radiation.

We detected a gradual increase in divergence among the population pairs evolving in parallel. A similar situation of sympatric differentiation is reported for the cichlid genus *Amphilophus*, which demonstrates a stage‐specific speciation process from incipient to more advanced stages of speciation (Sowersby et al., 2021; Torres‐Dowdall & Meyer, 2021). These examples correspond to the concept of the speciation continuum that is considered a speciation as a continuum of stages of reproductive (and other) isolations (Bolnick et al., 2023; Drès & Mallet, 2002; Stankowski & Ravinet, 2021). It is noteworthy that such a continuum is found in *Labeobarbus* populations inhabiting the rivers while all previous examples (among fishes) came from the lacustrine environment. Generally, ecological speciation is more common among fishes under the lacustrine conditions (reviewed in Seehausen & Wagner, 2014). However, the Ethiopian cyprinids (Golubtsov et al., 2021; Levin et al., 2019, 2020, 2021) and South American cichlids (Burress et al., 2018), as well as the Kamchatka salmonids (Esin et al., 2021, 2022), demonstrate this evolutionary pattern in rivers.

For a better understanding of the nature of numerously proliferated polymorphisms of the mouth structure in *Labeobarbus*, we have to study the evolutionary history of the Torinae lineages in the future. Mouth polymorphisms in relation to lip size including the state “hyperthophied lips” is common within the closely related polyploid cyprinid lineages from the Middle East (e.g., *Arabibarbus* Borkenhagen, 2014 and *Carasobarbus* Karaman, 1971). This polymorphism is probably ancestral to both African and Middle East Torinae being inherited from Southern and Southeastern tetraploid Torinae lineages (e.g., *Tor* Gray, 1834 and *Neolissochilus* Rainboth, 1985 – Roberts & Khaironizam, 2008; Yang et al., 2015, 2022). Thus, a proliferation of a repeated and predicted thick‐lipped phenotype within *Labeobarbus* lineage might be due to an ancient genetic polymorphism that re‐evolved under particular ecological circumstances. Given this, the thick‐lipped phenotype of *Labeobarbus* is supposedly “pre‐adaptive” upon emergence *de‐novo* in various populations but not always functional, i.e., not yet necessarily involved in trophic resource partitioning according to our results (Figures 3b and 4). Nevertheless, the prevalence of invertebrates in a diet or elevated d^15^N values in the thick‐lipped ecomorph were discovered in all cases (including non‐significant differences). This finding may be interpreted as a result of the generally adaptive value of the hypertrophied lips. In those populations that could not demonstrate an adaptive value of the hypertrophied lips in terms of trophic resource partitioning, the thickened lips might serve as a latent “adaptation in reserve” or “silent adaptation” that can be recruited into invertebrate foraging at appropriate environmental conditions possible due to being maintained in the population in low frequency and due to simple genetic and developmental programs that might also be facilitated by genetic assimilation based on initially phenotypic plasticity (Gunter et al., 2017; Meyer, 1987; Schneider et al., 2014). This suggestion should be tested in future studies.

## 5 CONCLUSIONS

We showed that the repeated origin of sympatric ecomorphs differing in lip size within the East African *Labeobarbus* provides a nice example for studying ecological speciation. The six pairs of sympatric ecomorphs that have been equally divergent in phenotype (thick‐lipped vs. common lips) were at various stages of ecological and genetic differentiation. This example of ecological speciation represents a continuum from polymorphism in phenotype via trophic resource partitioning (with various strengths) to genetic divergence. This assumedly starts as an intra‐population strategy to broaden the food spectrum via temporally/seasonally differential niches. However, in some circumstances, polymorphism in lip size may be a major contributor to trophic shift and consequently to divergent selection and speciation, for example, in the Didessa River (see also example from cichlids – Elmer et al., 2010).

## AUTHOR CONTRIBUTIONS

**Boris Levin:** Conceptualization (equal); data curation (equal); formal analysis (equal); funding acquisition (equal); investigation (equal); methodology (equal); project administration (equal); resources (equal); supervision (equal); validation (equal); visualization (equal); writing – original draft (equal); writing – review and editing (equal). **Aleksandra Komarova:** Conceptualization (equal); data curation (equal); formal analysis (equal); investigation (equal); visualization (equal); writing – original draft (equal); writing – review and editing (equal). **Evgeniy Simonov:** Formal analysis (equal); investigation (equal); methodology (equal); validation (equal); visualization (equal); writing – original draft (equal); writing – review and editing (equal). **Alexei Tiunov:** Formal analysis (equal); investigation (equal); project administration (equal); writing – original draft (equal); writing – review and editing (equal). **Marina Levina:** Data curation (equal); formal analysis (equal); investigation (equal); writing – original draft (equal); writing – review and editing (equal). **Alexander Golubtsov:** Conceptualization (equal); investigation (equal); writing – original draft (equal); writing – review and editing (equal). **Fyodor Kondrashov:** Investigation (equal); resources (equal); writing – original draft (equal); writing – review and editing (equal). **Axel Meyer:** Conceptualization (equal); investigation (equal); resources (equal); supervision (equal); writing – original draft (equal); writing – review and editing (equal).

## DATA ACCESSIBILITY AND BENEFIT‐SHARING

We prepare our data to archive in a publicly accessible repository – in Dryad and GenBank, in particular.

## Supporting information


Appendix S1:

## Data Availability

Morphologic data, data on diet and stable isotope composition, mtDNA dataset (cytochrome *b*), and genotyping files (various sets of SNPs) have been uploaded to Dryad: https://doi.org/10.5061/dryad.rbnzs7hgn. Genetic (new cytochrome *b* sequences) and genomic data (raw reads) were deposited to GenBank under Accession numbers OQ604627–OQ604649, and Bioproject ID PRJNA1000117, respectively.
