# Supplementary material for: Speciation and repeated origins of hypertrophied lips in parallel adaptive radiations of cyprinid fish from East Africa
Source: Ecol Evol. 2023 Sep 12;13(9):e10523. doi: 10.1002/ece3.10523 (PMC10497736; doi:10.1002/ece3.10523)
Supplement: Supplementary file 1 — Appendix S1: [file ECE3-13-e10523-s001.zip › Supplementary materials to LIPPED_upd.docx]

**Supplementary materials to MS “A speciation continuum within repeated origin of hypertrophied lips in cyprinid fish from East Africa”**

by

Boris Levin, Aleksandra Komarova, Evgeniy Simonov, Alexei Tiunov, Marina Levina, Alexander Golubtsov, Fyodor Kondrashov, and Axel Meyer

**List of Supplementary materials**

1. **Supplementary Table S1.** Sample size of the generalized and thick-lipped ecomorphs of *Labeobarbus* for various analyses.
2. **Supplementary Figure S2.** Violin boxplots of standard length (SL) in sympatric ecomorphs *Labeobarbus* from the Birbir R., Blue Nile R., Didessa R., Genale R., Gojeb R., and Sore R.
3. **Supplementary Table S3**. NCBI numbers of mtDNA (cytochrome *b*) of thick-lipped and generalized ecomorphs of *Labeobarbus* spp. from the Ethiopian Highlands used in this study – File is presented as separated Excel File.
4. **Supplementary Table S4**. Eigenvectors of characters to Fig. 4A.
5. **Supplementary Material S5.** PCA of all ecomorphs from six rivers, loadings of PCA and eigenvectors of characters.
6. **Supplementary Figure S6**. (A) Violin plots of relative lower lip lobe lengths (*MLL*) in sympatric ecomorphs *Labeobarbus* from the Birbir R., Blue Nile R., Didessa R., Genale R., Gojeb R., and Sore R.; (B) Violin plots of relative gut length of sympatric ecomorphs *Labeobarbus* spp. from the same rivers.
7. **Supplementary material S7**. Detailed description of the diet of each ecomorph in certain rivers to Figure 5.
8. **Supplementary Table S8**. Basic statistics for both δ^15^N and δ^13^C values.
9. **Supplementary Figure S9** (to Figure 5). Biplots of stable isotope composition (means of δ15N and δ13C values ± SD) of the sympatric ecomorphs of the *Labeobarbus* spp. from the Birbir, Blue Nile, Didessa, Genale, Gojeb, and Sore Rivers.
10. **Supplementary Table S10**. Estimated isotopic niche areas of the sympatric ecomorphs of the *Labeobarbus* spp. from the Birbir, Blue Nile, Didessa, Genale, Gojeb, and Sore.
11. **Supplementary Table S11**. Niche overlap estimates (NicheROVER package) showing the posterior probabilities (α = 0.95) that individuals from rows will be found within the niches indicated by the column header.
12. **Supplementary Table S12.** *P*-distances between geographic populations of *Labeobarbus* from drainages of Ethiopian Highlands estimated by cytochrome *b* sequences (1038 bp).
13. **Supplementary File S13**. Raw statistics on reads to ddRAD sequencing.
14. **Supplementary File S14**. Reich *F*_ST_ pairwise comparisons between inferred genetic clusters.
15. **Supplementary File S15**. Reich *F*_ST_ pairwise comparisons of the pairs of sympatric ecomorphs within each basin.

**Table S1.** Sample size of the generalized and thick-lipped ecomorphs of *Labeobarbus* for various analyses.

| **Ecomorphs** | **Morphology** | **Gut Length** | **Diet** | **Stable Isotopes** | **mtDNA** | **ddRAD** |
| --- | --- | --- | --- | --- | --- | --- |
| **Birbir R.** | | | | | | |
| Generalized | 33 | 30 | – | 17 | 25 | 4 |
| Thick-lipped | 2 | 1 | – | 2 | 3 | 1 |
| **Blue Nile R.** | | | | | | |
| Generalized | 31 | 15 | 15 | 23 | 21 | - |
| Thick-lipped | 5 | 7 | 7 | 2 | 2 | - |
| **Didessa R.** | | | | | | |
| Generalized | 10 | 15 | 6 | 20 | 20 | 5 |
| Thick-lipped | 6 | 6 | 2 | 7 | 10 | 5 |
| **Genale R.** | | | | | | |
| Generalized | 25 | 38 | 12 | 21 | 32 | 22 |
| Thick-lipped | 13 | 24 | 8 | 14 | 17 | 8 |
| **Gojeb R**. | | | | | | |
| Generalized | 34 | 45 | 17 | 40 | 30 | 7 |
| Thick-lipped | 21 | 27 | 14 | 19 | 17 | 4 |
| **Sore R.** | | | | | | |
| Generalized | 59 | 75 | – | 59 | 25 | 1 |
| Thick-lipped | 13 | 13 | – | 13 | 11 | 6 |
|  | | | | | | |
| **Total** | **252** | **296** | **81** | **237** | **213** | **63** |

**Supplementary Figure S2.** Violin boxplots of standard length (SL) in sympatric ecomorphs *Labeobarbus* from the Birbir R., Blue Nile R., Didessa R., Genale R., Gojeb R., and Sore R. G – generalized, L – thick-lipped, Min-max values (whiskers), 1st and 3rd quartiles (white vertical bars), median values (black horizontal bars), and outliers (black points) are indicated. No significant differences (p<0.05) between sympatric ecomorphs (Wilcoxon test) or between all ecomorphs (Kraskell-Wallis test) were detected.


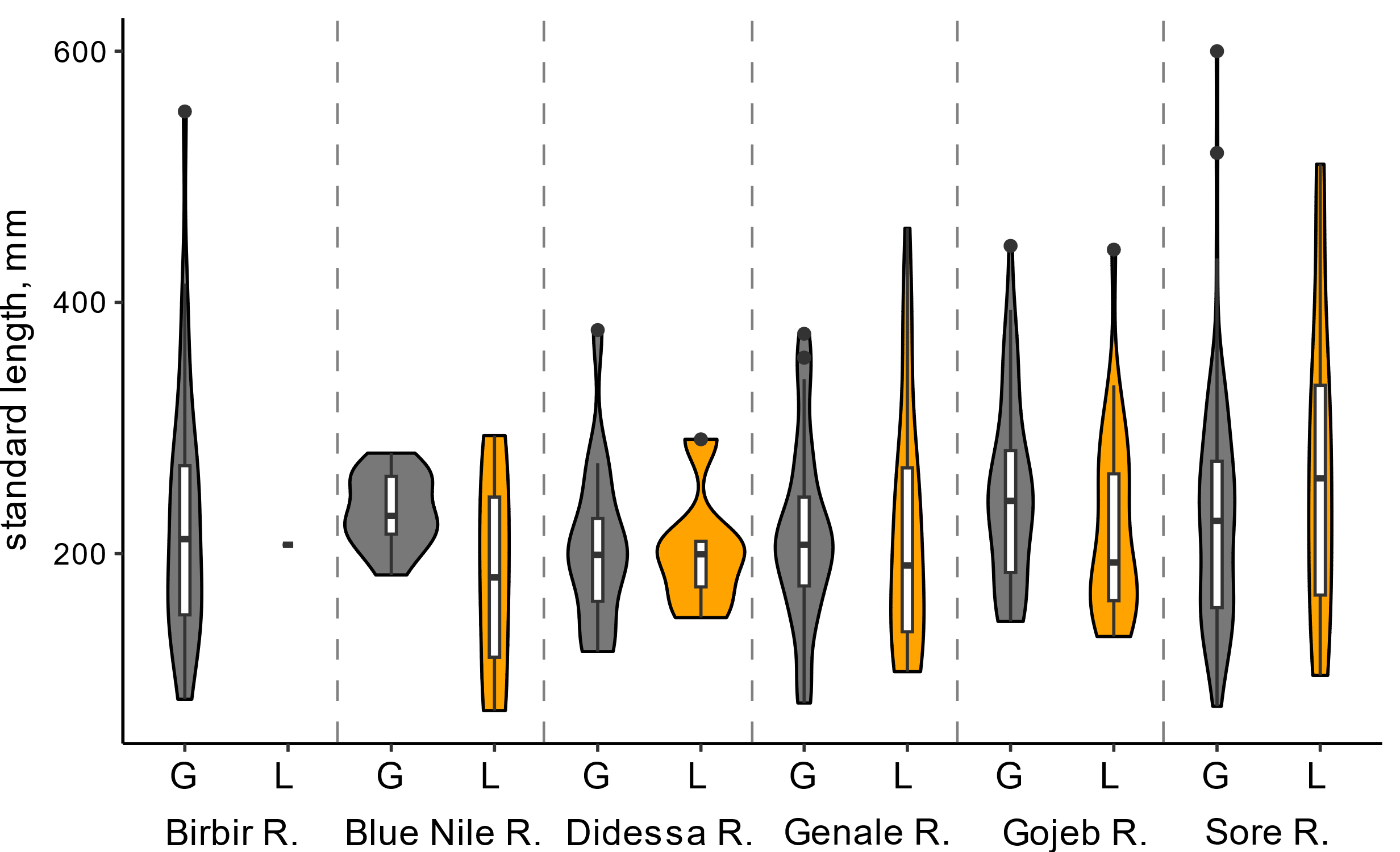


**Supplementary Table S3**. NCBI numbers of mtDNA (cytochrome *b*) of thick-lipped and generalized ecomorphs of *Labeobarbus* spp. from the Ethiopian Highlands used in this study – File is presented as separated Excel File.

**Supplementary Table S4**. Eigenvectors of characters to Fig. 4A.

| Characters/PCs | **Birbir R** | | **Blue Nile R.** | | **Didessa R** | | **Genale R** | | **Gojeb R** | | **Sore R** | |
| --- | --- | --- | --- | --- | --- | --- | --- | --- | --- | --- | --- | --- |
|  | PC1 | PC2 | PC1 | PC2 | PC1 | PC2 | PC1 | PC2 | PC1 | PC2 | PC1 | PC2 |
| MLL | 0.71 | 0.04 | 0.72 | 0.09 | 0.66 | -0.21 | 0.68 | -0.11 | -0.63 | 0.26 | 0.72 | -0.16 |
| LLL | 0.15 | 0.97 | 0.23 | 0.90 | 0.40 | 0.91 | 0.37 | 0.90 | -0.47 | -0.88 | 0.12 | -0.93 |
| ULW | 0.69 | -0.25 | 0.65 | -0.42 | 0.64 | -0.36 | 0.63 | -0.41 | -0.61 | 0.40 | 0.68 | 0.34 |

**Supplementary Material S5.** PCA of all ecomorphs from six rivers, loadings of PCA and eigenvectors of characters. G – generalized, L – thick-lipped.


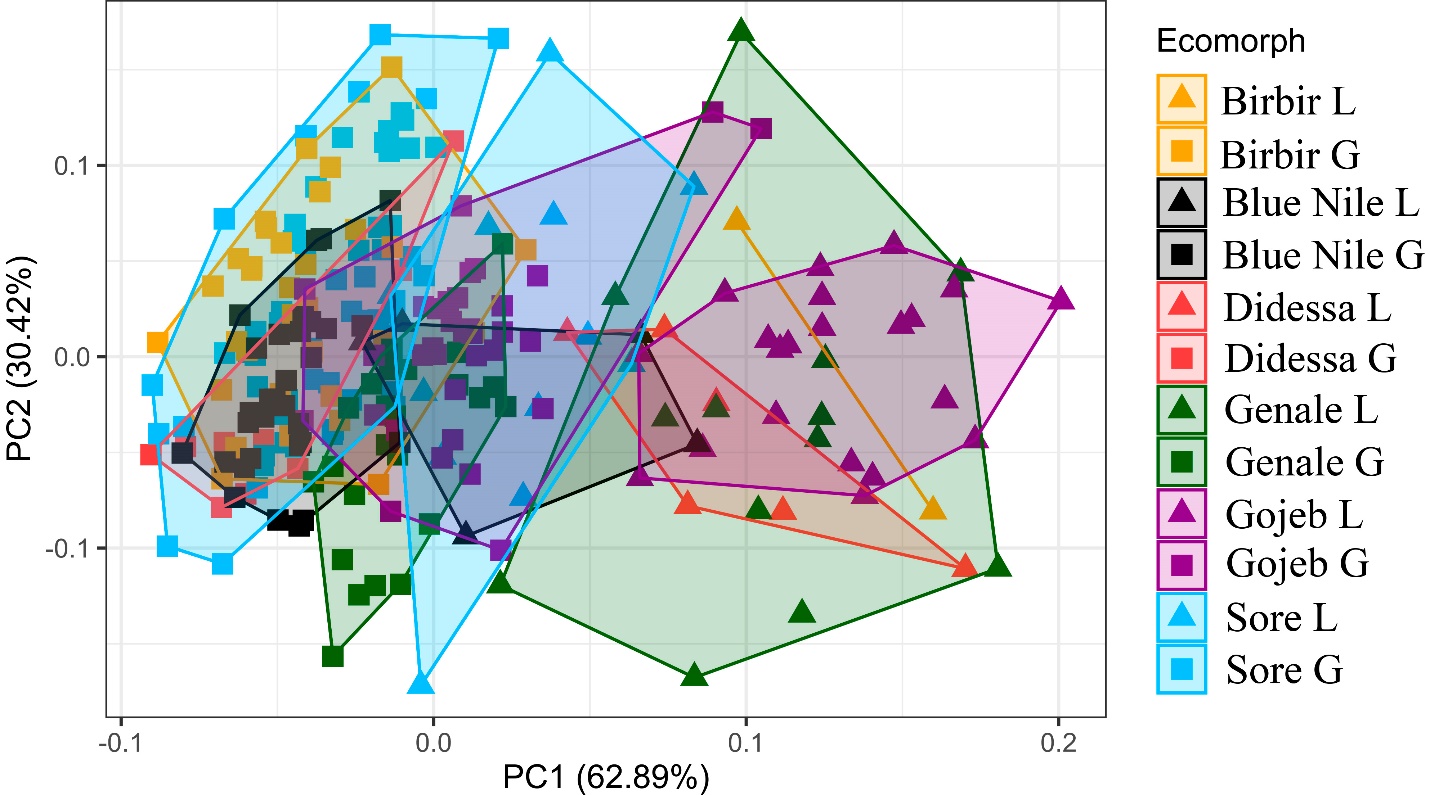


|  | eigenvalue | variance.percent | cumulative.variance.percent |
| --- | --- | --- | --- |
| Dim.1 | 1.8867849 | 62.892829 | 62.89283 |
| Dim.2 | 0.9125837 | 30.419457 | 93.31229 |
| Dim.3 | 0.2006314 | 6.687714 | 100.00000 |
|  |  |  |  |
|  |  |  |  |
|  | PC1 | PC2 | PC3 |
| MLL | 0.6836659 | -0.1257712 | 0.7188759 |
| LLL | 0.3240622 | 0.9349162 | -0.1446213 |
| ULW | 0.6538995 | -0.3318331 | -0.6799281 |

**Supplementary Figure S6**. Violin plots of relative lower lip middle lobe lengths (*MLL*) in sympatric ecomorphs *Labeobarbus* from the Birbir R., Blue Nile R., Didessa R., Genale R., Gojeb R., and Sore R.; B) Violin plots of relative gut length of sympatric ecomorphs *Labeobarbus* spp. from the same rivers. G – generalized, L – thick-lipped, Min-max values (whiskers), 1st and 3rd quartiles (white vertical bars), median values (black horizontal bars), and outliers (black points) are indicated. The lowercase letters above the violin plots indicate significant differences between sympatric ecomorphs (p < 0.01. Wilcoxon test).


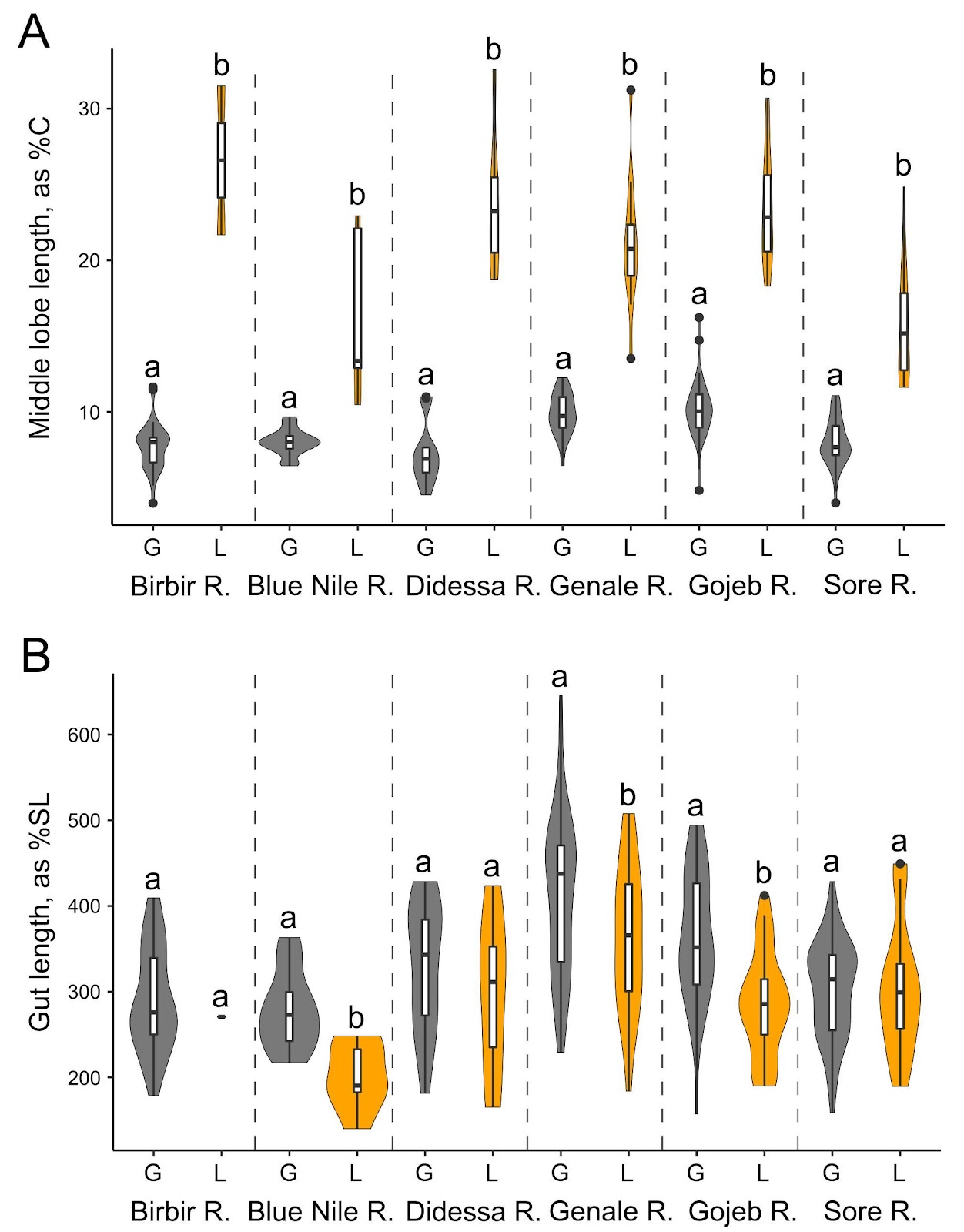


**Supplementary material S7**. Detailed description of the diet of each ecomorph in certain rivers to Figure 5.

*Blue Nile River.* The guts were half-full, both generalized and thick-lipped ecomorphs had an average fullness of 67.8% and 41%, respectively. In regarding to the generalized ecomorphs, detritus significantly predominated (IR = 83.1%) while invertebrates (IR = 9.0%) and macrophytes (IR = 4.0%) were less represented. Invertebrates were detected in all guts of generalized ecomorph fishes and were represented by the larvae of Ephemeroptera, Trichoptera, and Diptera (Ceratopogonidae and Chironomidae), and imagoes of Diptera. Portion of sand was < 5% by mass. Five guts contained scales of fishes (<1% by mass). Regarding to the hypertrophied thick-lipped ecomorph, a detritus was also the main food item (IR =69.1%) while portion of invertebrates (IR = 20.2%) was twice larger. Macrophytes were hardly represented (IR = 1.6%). Invertebrates were represented by the larvae of Ephemeroptera, Trichoptera, and Diptera (Simulidae, Ceratopogonidae, and Chironomidae), and imago of Diptera. One gut contained remnants of fish. Portion of sand was < 8.7% by mass.

*Didessa River*. The guts were rather full, both generalized and thick-lipped ecomorphs had fullness of on average 81.3% and 81.5%, respectively. Regarding to generalized ecomorph, detritus significantly predominated (IR = 81.1%) while invertebrates (IR = 11.4%) and macrophytes (IR = 7.4%) were less represented. Invertebrates were detected in all guts of generalized ecomorph and were represented by larvae of Ephemeroptera, Trichoptera, and Diptera (Simuliidae, Ceratopogonidae, and Chironomidae), and imago of Hemiptera, Coleoptera, Hymenoptera, and Diptera. Three guts contained scales of fishes. Portion of sand was < 1% by mass. The thick-lipped ecomorph consumed more invertebrates (IR = 35.5%) than generalized ecomorph but detritus is still dominating food item (IR = 59.4%). Invertebrates were represented by larvae of Ephemeroptera, Trichoptera, Coleoptera, and Diptera (Ceratopogonidae. Chironomidae) as well as imago of Hymenoptera. One gut contained a few individuals of Cladocera. Macrophytes contribution was low (IR = 3.4%). Portion of sand was < 2% by mass

*Genale River*. The guts were almost half-full, both generalized and thick-lipped ecomorphs had fullness 64.4% and 57.7%, respectively. One gut of thick-lipped ecomorph was empty. Regarding to IR the generalized ecomorph, a detritus was the main food item (IR = 92.1%) while macrophytes (IR = 7.5%) and invertebrates (IR < 1%) were significantly less represented. At the same time invertebrates (insects) were detected in all guts and were represented by larvae of Ephemeroptera, Trichoptera, Coleoptera, and Diptera (Ceratopogonidae, Chironomidae) as well as imagoes of Hemiptera and Diptera. One gut contained Copepoda singly. Portion of sand did not exceed 1% by mass. Regarding to a thick-lipped ecomorph, a detritus was also the main food item (IR = 79.0%) while macrophytes (IR = 9.2%) and invertebrates (IR = 9.0%) were significantly less represented. Invertebrates were represented by larvae of Ephemeroptera, Trichoptera, Coleoptera, and Diptera (Ceratopogonidae, Chironomidae), and imagoes of Coleoptera and Hymenoptera. Single individuals of Cladocera were recorded in one gut. Two guts contained fish scales (<1% by mass). Portion of sand was < 4% by mass.

*Gojeb River*. The guts were filled partially - generalized and thick-lipped ecomorphs had average fullness of 25.2% and 33.6%, respectively. Regarding to generalized ecomorph, a detritus was the main food item (IR = 63.8%); contribution of macrophytes (IR = 20.6%) and invertebrates (IR = 14.1%) were also remarkable. Invertebrates were detected in all guts and were represented by larvae of Ephemeroptera, Trichoptera, Coleoptera, and Diptera (Ceratopogonidae, Chironomidae) as well as imago of Hemiptera, Coleoptera, and Hymenoptera. One gut contained a few remnants of fish (vertebrae and other bones) having <1% by mass from other food items. Two guts contained scales of fishes (<1% by mass). Proportion of sand was < 2% by mass. Regarding to the thick-lipped ecomorph, invertebrates dominated among other items (IR = 53.5%) and were represented by larvae of Ephemeroptera, Trichoptera, Coleoptera, and Diptera (Ceratopogonidae, Chironomidae) as well as imagoes of Hemiptera, Coleoptera, and Hymenoptera. Contribution of detritus (IR = 27.1%) and macrophytes (IR = 18.0%) was remarkably fewer. One gut contained Cladocera singly. Three guts contained fish scales (<1% by mass). The portion of sand was < 2% by mass.

**Supplementary Table S8**. Basic statistics for both δ^15^N and δ^13^C values - is given separately as an Excel File.

**Supplementary Figure S9** (to Figure 5). Biplots of stable isotope composition (means of δ15N and δ13C values ± SD) of the sympatric ecomorphs of the *Labeobarbus* spp. from the Birbir, Blue Nile, Didessa, Genale, Gojeb, and Sore Rivers.


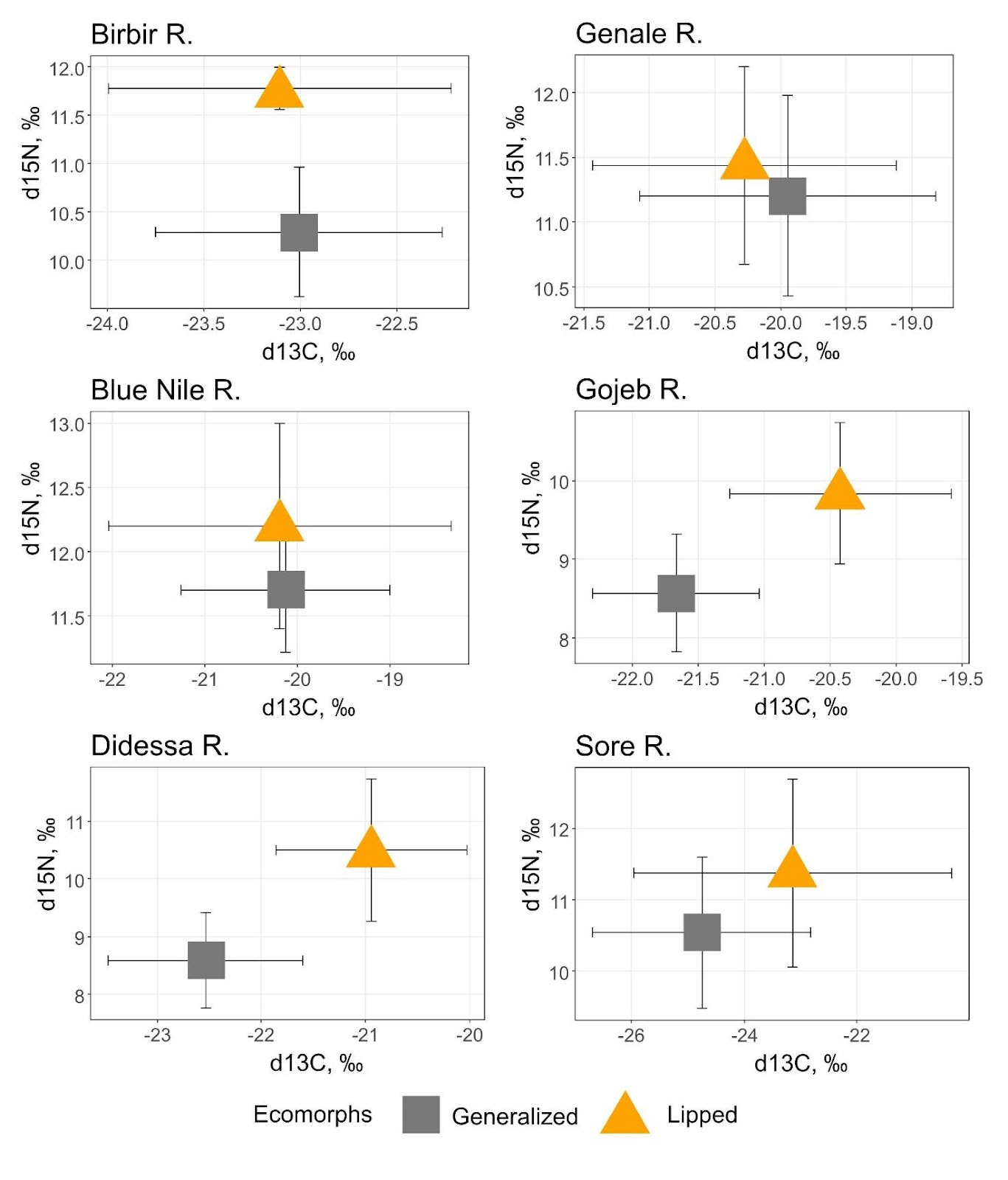


**Supplementary Table S10**. Estimated isotopic niche areas of the sympatric ecomorphs of the *Labeobarbus* spp. from the Birbir, Blue Nile, Didessa, Genale, Gojeb, and Sore. TA. SEA. and SEAc (SIBER package): Total area of convex hull. standard ellipse area. and corrected standard ellipse area with a correction for small sample sizes. respectively. G – generalized. L – thick-lipped.

|  | Birbir | | Blue Nile | | Didessa | | Genale | | Gojeb | | Sore | |
| --- | --- | --- | --- | --- | --- | --- | --- | --- | --- | --- | --- | --- |
|  | G  (17) | L  (2) | G  (23) | L  (2) | G  (20) | L  (7) | G  (21) | L  (14) | G  (40) | L  (19) | G  (59) | L  (13) |
| TA | 3.49 | – | 5.21 | – | 6.1 | 4.25 | 8.13 | 3.98 | 5.35 | 5.29 | 22.6 | 8.93 |
| SEA | 1.42 | – | 1.56 | – | 1.97 | 3.16 | 2.64 | 2.03 | 1.42 | 1.94 | 3.57 | 3.85 |
| SEAc | 1.51 | – | 1.64 | – | 2.08 | 3.8 | 2.78 | 2.2 | 1.46 | 2.06 | 3.63 | 4.2 |

**Supplementary Table S11**. Niche overlap estimates (NicheROVER package) showing the posterior probabilities (α = 0.95) that individuals from rows will be found within the niches indicated by the column header. Results (%) are provided for sympatric ecomorphs of the *Labeobarbus* spp.

| **Didessa R.** | Generalized | Lipped |
| --- | --- | --- |
| Generalized | NA | 24.95 |
| Lipped | 26.37 | NA |
| **Genale R.** | Generalized | Lipped |
| Generalized | NA | 74.72 |
| Lipped | 89.92 | NA |
| **Gojeb R.** | Generalized | Lipped |
| Generalized | NA | 69.13 |
| Lipped | 45.26 | NA |
| **Sore R.** | Generalized | Lipped |
| Generalized | NA | 82.34 |
| Lipped | 80.88 | NA |

**Supplementary Table S12**. *P*-distances between geographic populations of *Labeobarbus* from drainages of Ethiopian Highlands estimated by cytochrome *b* sequences (1038 bp).

| **Group 1** | **Group 2** | **P-distance** | **SE** |
| --- | --- | --- | --- |
|  |  |  |  |
| Birbir | Didessa | 0.014 | 0.003 |
| Birbir | Genale | 0.025 | 0.004 |
| Didessa | Genale | 0.028 | 0.005 |
| Birbir | Gojeb | 0.009 | 0.003 |
| Didessa | Gojeb | 0.012 | 0.003 |
| Genale | Gojeb | 0.025 | 0.004 |
| Birbir | Blue Nile | 0.014 | 0.003 |
| Didessa | Blue Nile | 0.003 | 0.001 |
| Genale | Blue Nile | 0.028 | 0.005 |
| Gojeb | Blue Nile | 0.013 | 0.003 |
| Birbir | Sore | 0.019 | 0.004 |
| Didessa | Sore | 0.019 | 0.004 |
| Genale | Sore | 0.031 | 0.005 |
| Gojeb | Sore | 0.019 | 0.004 |
| Blue Nile | Sore | 0.019 | 0.004 |

**Supplementary File S13**. Raw statistics on reads to ddRAD sequencing – is given separately as Excel File S10.

**Supplementary File S14**. Reich *F*_ST_ pairwise comparisons between inferred genetic clusters.

Pairwise Reich F_ST_ estimates with 95% CI based on 1000 bootstraps

Plot


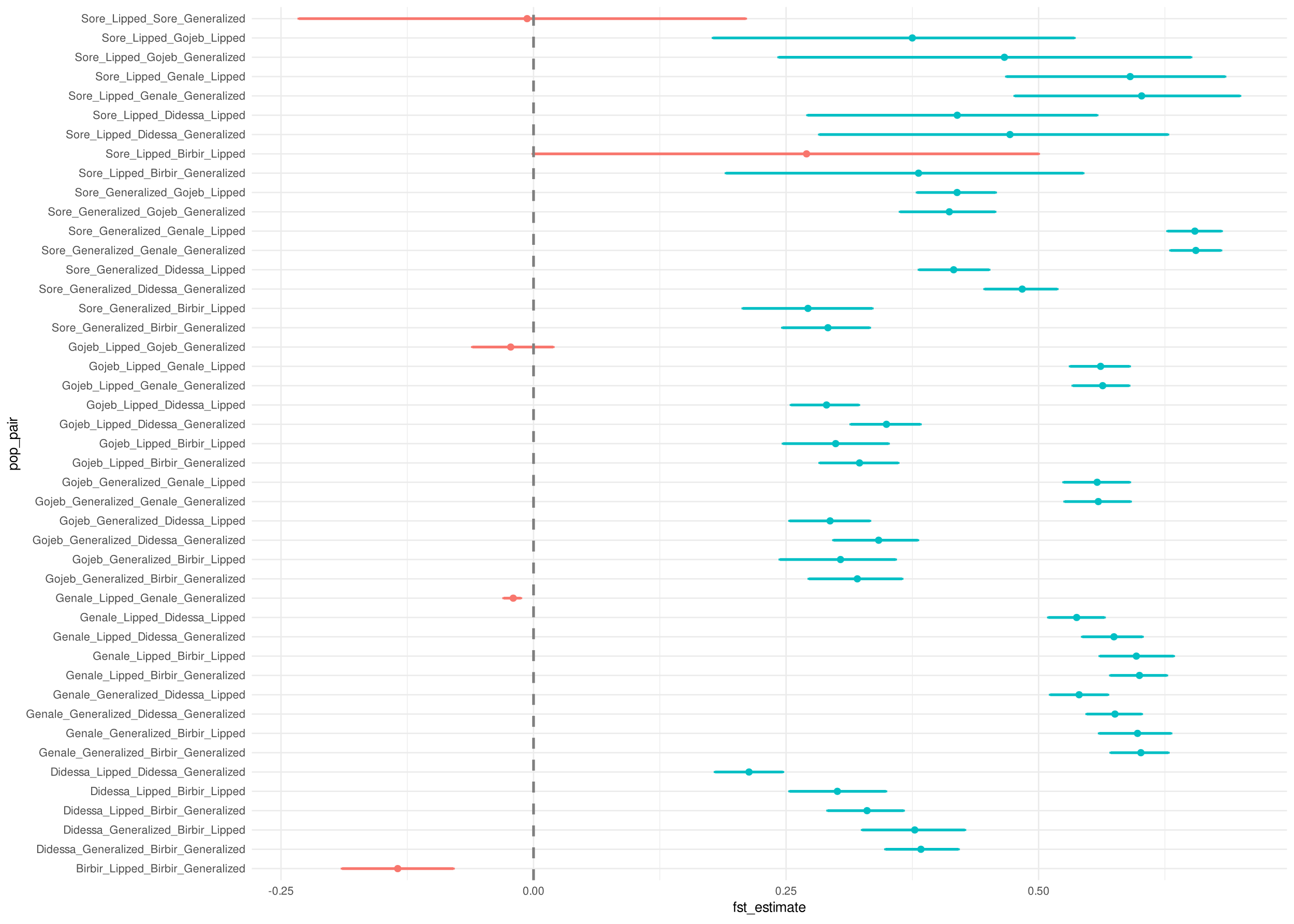


Table

| **pop1** | **pop2** | **fst_estimate** | **min_CI** | **max_CI** |
| --- | --- | --- | --- | --- |
| Birbir_Lipped | Birbir_Generalized | -0.13444366252248 | -0.1895571 | -0.0793303 |
| **Didessa_Generalized** | **Birbir_Generalized** | **0.383510031039443** | **0.3484118** | **0.4206311** |
| **Didessa_Lipped** | **Birbir_Generalized** | **0.330147022881692** | **0.2911266** | **0.3662618** |
| **Genale_Generalized** | **Birbir_Generalized** | **0.601247499722712** | **0.5714289** | **0.6283747** |
| **Genale_Lipped** | **Birbir_Generalized** | **0.599829764517935** | **0.5710544** | **0.6268604** |
| **Gojeb_Generalized** | **Birbir_Generalized** | **0.32053772070626** | **0.2725647** | **0.3648284** |
| **Gojeb_Lipped** | **Birbir_Generalized** | **0.322707733377654** | **0.2833276** | **0.3609362** |
| **Sore_Generalized** | **Birbir_Generalized** | **0.291429608770728** | **0.2463913** | **0.3327488** |
| **Sore_Lipped** | **Birbir_Generalized** | **0.381124290871583** | **0.1905644** | **0.5440635** |
| **Didessa_Generalized** | **Birbir_Lipped** | **0.377356347944583** | **0.3253807** | **0.4269939** |
| **Didessa_Lipped** | **Birbir_Lipped** | **0.300783707329348** | **0.2535105** | **0.3488099** |
| **Genale_Generalized** | **Birbir_Lipped** | **0.597929946270445** | **0.5599731** | **0.6310859** |
| **Genale_Lipped** | **Birbir_Lipped** | **0.596757233627105** | **0.5606337** | **0.6336705** |
| **Gojeb_Generalized** | **Birbir_Lipped** | **0.303904938598816** | **0.2438725** | **0.3584538** |
| **Gojeb_Lipped** | **Birbir_Lipped** | **0.299089842875674** | **0.2470321** | **0.3513577** |
| **Sore_Generalized** | **Birbir_Lipped** | **0.271666911054451** | **0.2072003** | **0.3353047** |
| Sore_Lipped | Birbir_Lipped | 0.27027027027027 | -0.0005814 | 0.5000000 |
| **Didessa_Lipped** | **Didessa_Generalized** | **0.213303291053441** | **0.1796927** | **0.2467846** |
| **Genale_Generalized** | **Didessa_Generalized** | **0.57558007105171** | **0.5476483** | **0.6019866** |
| **Genale_Lipped** | **Didessa_Generalized** | **0.574580483180006** | **0.5432641** | **0.6027701** |
| **Gojeb_Generalized** | **Didessa_Generalized** | **0.341644991974418** | **0.2969541** | **0.3804225** |
| **Gojeb_Lipped** | **Didessa_Generalized** | **0.349349736482248** | **0.3140669** | **0.3829604** |
| **Sore_Generalized** | **Didessa_Generalized** | **0.483760760295967** | **0.4465800** | **0.5184052** |
| **Sore_Lipped** | **Didessa_Generalized** | **0.471620617631804** | **0.2829884** | **0.6280078** |
| **Genale_Generalized** | **Didessa_Lipped** | **0.540083396199103** | **0.5115278** | **0.5685986** |
| **Genale_Lipped** | **Didessa_Lipped** | **0.537656793003392** | **0.5096509** | **0.5651408** |
| **Gojeb_Generalized** | **Didessa_Lipped** | **0.293566642506026** | **0.2535278** | **0.3329543** |
| **Gojeb_Lipped** | **Didessa_Lipped** | **0.290036198489931** | **0.2549148** | **0.3219739** |
| **Sore_Generalized** | **Didessa_Lipped** | **0.415896800289308** | **0.3815897** | **0.4509496** |
| **Sore_Lipped** | **Didessa_Lipped** | **0.41944260302729** | **0.2712548** | **0.5579012** |
| Genale_Lipped | Genale_Generalized | -0.020097891204532 | -0.0295735 | -0.0125801 |
| **Gojeb_Generalized** | **Genale_Generalized** | **0.559074763810208** | **0.5258124** | **0.5909730** |
| **Gojeb_Lipped** | **Genale_Generalized** | **0.563418387825237** | **0.5339122** | **0.5895654** |
| **Sore_Generalized** | **Genale_Generalized** | **0.655643104230359** | **0.6306101** | **0.6804666** |
| **Sore_Lipped** | **Genale_Generalized** | **0.601976462050263** | **0.4764117** | **0.6992856** |
| **Gojeb_Generalized** | **Genale_Lipped** | **0.557944259479069** | **0.5247807** | **0.5902207** |
| **Gojeb_Lipped** | **Genale_Lipped** | **0.56135480105906** | **0.5311961** | **0.5900952** |
| **Sore_Generalized** | **Genale_Lipped** | **0.654681192094864** | **0.6277078** | **0.6810555** |
| **Sore_Lipped** | **Genale_Lipped** | **0.590628743630196** | **0.4681120** | **0.6844903** |
| Gojeb_Lipped | Gojeb_Generalized | -0.0226120380101578 | -0.0605619 | 0.0195222 |
| **Sore_Generalized** | **Gojeb_Generalized** | **0.411587075426145** | **0.3628959** | **0.4568686** |
| **Sore_Lipped** | **Gojeb_Generalized** | **0.466125179168657** | **0.2426637** | **0.6508310** |
| **Sore_Generalized** | **Gojeb_Lipped** | **0.419285987998914** | **0.3797176** | **0.4573918** |
| **Sore_Lipped** | **Gojeb_Lipped** | **0.374947445478172** | **0.1777163** | **0.5353702** |
| Sore_Lipped | Sore_Generalized | -0.00634844787275798 | -0.2323141 | 0.2099888 |

Matrix of pairwise Reich F_ST_ estimates

|  | **Birbir_Generalized** | **Birbir_Lipped** | **Didessa_Generalized** | **Didessa_Lipped** | **Genale_Generalized** | **Genale_Lipped** | **Gojeb_Generalized** | **Gojeb_Lipped** | **Sore_Generalized** | **Sore_Lipped** |
| --- | --- | --- | --- | --- | --- | --- | --- | --- | --- | --- |
| Birbir_Generalized | NA | NA | NA | NA | NA | NA | NA | NA | NA | NA |
| Birbir_Lipped | 0.0000000 | NA | NA | NA | NA | NA | NA | NA | NA | NA |
| Didessa_Generalized | 0.3835100 | 0.3773563 | NA | NA | NA | NA | NA | NA | NA | NA |
| Didessa_Lipped | 0.3301470 | 0.3007837 | 0.2133033 | NA | NA | NA | NA | NA | NA | NA |
| Genale_Generalized | 0.6012475 | 0.5979299 | 0.5755801 | 0.5400834 | NA | NA | NA | NA | NA | NA |
| Genale_Lipped | 0.5998298 | 0.5967572 | 0.5745805 | 0.5376568 | 0.0000000 | NA | NA | NA | NA | NA |
| Gojeb_Generalized | 0.3205377 | 0.3039049 | 0.3416450 | 0.2935666 | 0.5590748 | 0.5579443 | NA | NA | NA | NA |
| Gojeb_Lipped | 0.3227077 | 0.2990898 | 0.3493497 | 0.2900362 | 0.5634184 | 0.5613548 | 0.0000000 | NA | NA | NA |
| Sore_Generalized | 0.2914296 | 0.2716669 | 0.4837608 | 0.4158968 | 0.6556431 | 0.6546812 | 0.4115871 | 0.4192860 | NA | NA |
| Sore_Lipped | 0.3811243 | 0.2702703 | 0.4716206 | 0.4194426 | 0.6019765 | 0.5906287 | 0.4661252 | 0.3749474 | 0 | NA |

Bibliography for R packages

Gruber, Bernd, Arthur Georges, Jose L. Mijangos, and Carlo Pacioni. 2022. *dartR: Importing and Analysing SNP and Silicodart Data Generated by Genome-Wide Restriction Fragment Analysis*. <https://CRAN.R-project.org/package=dartR>.

Gruber, Bernd, Peter J Unmack, Oliver F Berry, and Arthur Georges. 2018. “Dartr: An r Package to Facilitate Analysis of SNP Data Generated from Reduced Representation Genome Sequencing.” *Molecular Ecology Resources* 18: 691–99. <https://doi.org/10.1111/1755-0998.12745>.

Kamvar, Zhian N., Jonah C. Brooks, and Niklaus J. Grünwald. 2015. “Novel R tools for analysis of genome-wide population genetic data with emphasis on clonality.” *Front. Genet.* 6 (June): 208. <https://doi.org/10.3389/fgene.2015.00208>.

Kamvar, Zhian N., Javier F. Tabima, Jonah C. Brooks, and David Folarin. 2021. *Poppr: Genetic Analysis of Populations with Mixed Reproduction*. <https://CRAN.R-project.org/package=poppr>.

Kamvar, Zhian N., Javier F. Tabima, and Niklaus J. Grünwald. 2014. “*Poppr*: An R Package for Genetic Analysis of Populations with Clonal, Partially Clonal, and/or Sexual Reproduction.” *PeerJ* 2 (March): e281. <https://doi.org/10.7717/peerj.281>.

Knaus, Brian J., and Niklaus J. Grunwald. 2022. *vcfR: Manipulate and Visualize VCF Data*. <https://CRAN.R-project.org/package=vcfR>.

Knaus, Brian J., and Niklaus J. Grünwald. 2016. “VcfR: An r Package to Manipulate and Visualize VCF Format Data.” *BioRxiv*. <http://dx.doi.org/10.1101/041277>.

———. 2017. “VCFR: A Package to Manipulate and Visualize Variant Call Format Data in R.” *Molecular Ecology Resources* 17 (1): 44–53. <http://dx.doi.org/10.1111/1755-0998.12549>.

**Supplementary File S15**. Reich *F*_ST_ pairwise comparisons of the pairs of sympatric ecomorphs within each basin.

Plot


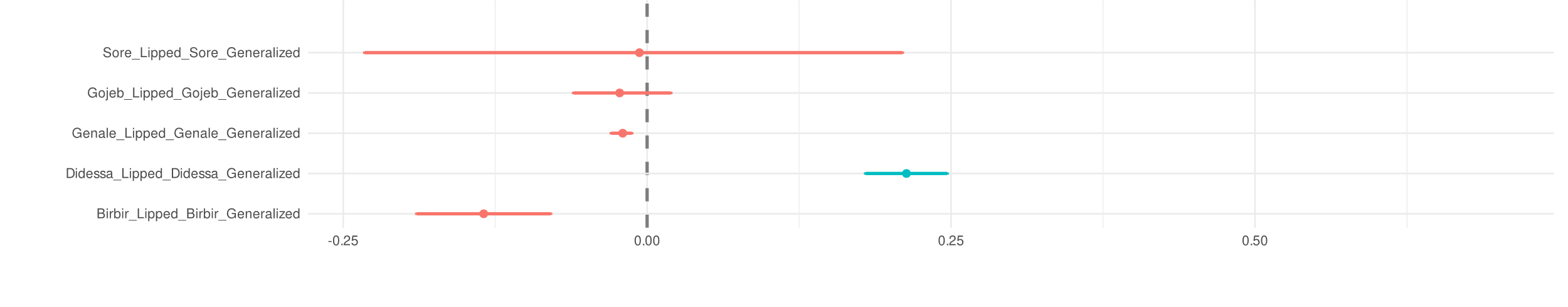


Note: the value of Reich *F*_ST_ pairwise comparison between Generalized and Thick-lipped ecomorphs in the Didessa River is 0.21 (taken from Table incorporated into Supplementary File S14 - see above).
